# The lncRNA BORG Drives Breast Cancer Metastasis and Disease Recurrence

**DOI:** 10.1038/s41598-017-12716-6

**Published:** 2017-10-05

**Authors:** Alex J. Gooding, Bing Zhang, Fereshteh Kenari Jahanbani, Hannah L. Gilmore, Jenny C. Chang, Saba Valadkhan, William P. Schiemann

**Affiliations:** 1 Case Comprehensive Cancer Center, Case Western Reserve University, Cleveland, OH 44106 USA; 2 Department of Molecular Biology and Microbiology, Case Western Reserve University, Cleveland, OH 44106 USA; 3 Department of Pathology, University Hospitals, Case Medical Center and Case Western Reserve University, Cleveland, OH 44106 USA; 4 Houston Methodist Research Center, Houston, TX 77030 USA

## Abstract

Long noncoding RNAs (lncRNAs) have emerged as potent regulators of breast cancer development and progression, including the metastatic spread of disease. Through *in silico* and biological analyses, we identified a novel lncRNA, **B**MP/**O**P-**R**esponsive **G**ene (BORG), whose expression directly correlates with aggressive breast cancer phenotypes, as well as with metastatic competence and disease recurrence in multiple clinical cohorts. Mechanistically, BORG elicits the metastatic outgrowth of latent breast cancer cells by promoting the localization and transcriptional repressive activity of TRIM28, which binds BORG and induces substantial alterations in carcinoma proliferation and survival. Moreover, inhibiting BORG expression in metastatic breast cancer cells impedes their metastatic colonization of the lungs of mice, implying that BORG acts as a novel driver of the genetic and epigenetic alterations that underlie the acquisition of metastatic and recurrent phenotypes by breast cancer cells.

## Introduction

Breast cancer remains the most frequently diagnosed malignancy and second most common cause of cancer-related death amongst females^1^. Complicating the diagnosis and clinical management of this disease is the propensity for breast cancers to shed malignant cells into the circulation during the earliest stages of tumor development^2,3^, resulting in the formation of metastatic lesions that are directly responsible for ~90% of breast cancer-related mortality^4,5^. Knowledge defining the precise genetic and epigenetic mechanisms governing the establishment and malignant progression of these deadly, frequently occult lesions is notably scarce and must be expanded in order to develop therapeutic strategies tailored specifically to inhibit the outgrowth of disseminated breast cancer cells.

Recent genomic analyses identified a new class of RNA called long noncoding RNA (lncRNA), whose members are broadly defined as RNA molecules greater than 200 nucleotides in length that lack an open reading frame capable of producing functional proteins^6^. Although originally believed to be transcriptional “noise”, emerging evidence now indicates that lncRNAs are expressed in a highly cell- and tissue-specific manner^7^. Moreover, lncRNAs form complex interactions with cellular proteins and chromatin architectural structures, a feature that endows these unique transcripts with numerous molecular functions^8,9^. Amongst these are the potent capacity of lncRNAs to alter the proliferative, invasive, and metastatic competency of malignant cells^10,11^, thus establishing oncogenic lncRNAs as potential therapeutic targets whose inhibition has demonstrated substantial efficacy in preclinical settings of metastatic disease^12,13^.

In this study, we scrutinized the function of the novel oncogenic lncRNA, **B**MP/**O**P-**R**esponsive **G**ene (BORG)^14^, particularly its ability to alter the proliferation and survival of disseminated breast cancer cells. Our analyses reveal that high BORG expression is tightly linked to metastatic and recurrent disease, making it a potentially robust biomarker capable of identifying women at high risk for developing invasive, metastatic, and recurrent breast cancers. We further show that the oncogenic activities of BORG are mediated in part through its binding to the E3 SUMO ligase, TRIM28 (KAP1), which functions as a (i) transcriptional co-repressor and scaffolding protein for histone and DNA modifying enzymes; and (ii) stimulator of breast cancer proliferation by suppressing the transcription of *Cdkn1a* (*p21*) and *Gadd45a* ^15–18^. Importantly, the formation of BORG:TRIM28 complexes was sufficient to drive latent disseminated breast cancer cells to reactivate proliferative programs coupled to disease recurrence, thereby establishing BORG, TRIM28, and their downstream effectors as clinically relevant targets to alleviate breast cancer metastasis.

## Results

### BORG expression correlates with the malignant and metastatic potential of breast cancers

Despite the clinical burden associated with metastasis, the identification and characterization of the molecular mediators (*e.g*., lncRNAs) vital to fostering metastatic outgrowth remains incomplete. As such, we conducted an *in silico* investigation of publically available RNA-seq datasets to identify novel lncRNAs associated with malignant and metastatic progression of mammary tumors. In doing so, we used the Mann-Whitney U-test (coefficient ≤0.005) to identify intergenic lncRNAs that showed >2-fold overexpression in triple-negative breast cancer (TNBC) tissues *versus* those expressed in their normal tissue counterparts^19,20^, as well as lncRNAs that were upregulated >2-fold in high-grade pre-invasive breast cancers as compared to their adjacent normal tissue^19,21^. Lastly, we identified lncRNAs that exhibited >1.5-fold overexpression in the primary breast tumors of patients who presented with metastatic disease within 5 years of their initial diagnosis and treatment *versus* lesions arising in patients who maintained greater than 5 years of disease-free survival following their primary treatment^22^. lncRNAs that were expressed at a minimum abundance of 0.3 transcripts per million cellular transcripts were included in the analysis and summarized in Table 1.

**Table 1:** lncRNAs upregulated in malignant and metastatic breast cancers *versus* indolent counterparts.

lncRNA name	PRJNA227137 (TNBC vs normal)	PRJNA251383 (TNBC vs normal)	PRJNA284949 (DCIS vs normal)	PRJNA284949 (High grade vs low grade DCIS)	PRJEB9083 (High vs low metastatic risk TNBC)
LINC01562	6.479798288	2.070056809	3.179928008	2.067772964	1.671722876
RP1-78B3.1	9.944502997	2.267231205	3.113076321	2.77166513	1.525467872
RP11-160H22.5	10.0854205	2.216582873	2.447095437	2.002746383	1.646468015
HCG20	10.47546451	2.759449663	5.823163757	4.806079042	1.875749504
AC009502.4	10.84962373	2.040056285	2.454486569	2.602690419	3.114086
SGO1-AS1	11.00801689	2.337434241	2.786293423	6.81361168	1.570663863
RP13-631K18.2	11.35403291	2.087397651	2.430895751	2.042671516	1.891258535
BORG	12.66047092	2.038470142	2.019287548	2.114221168	1.864568167
AP000476.1	14.027197	3.025196601	3.310936602	2.995635908	1.502131509
LINC01206	17.177809	2.002136506	3.001076225	2.464426329	1.641174865
RP11-322E11.2	17.92682739	2.071539228	2.763097147	2.948282083	1.718026234
PRNCR1	18.88104658	2.325914985	2.517058334	2.564753369	2.081802874
MIR663AHG	19.59234597	2.597594979	8.306125226	9.531079355	2.885357579
RP11-321L2.2	21.29775567	2.613644314	2.819629629	3.473131593	1.762116467
RP11-615I2.6	25	2.209056388	2.978720525	2.064556924	1.677629554
RP11-116D17.4	25	2.426469084	3.394734009	2.728555094	1.697222802
RP11-326N17.2	25	2.677061954	2.70393967	2.779428924	1.563150597

TNBC: Triple-Negative Breast Cancer; DCIS: Ductal Carcinoma *in situ*; PRJN: Project accession number

**Figure 1:**
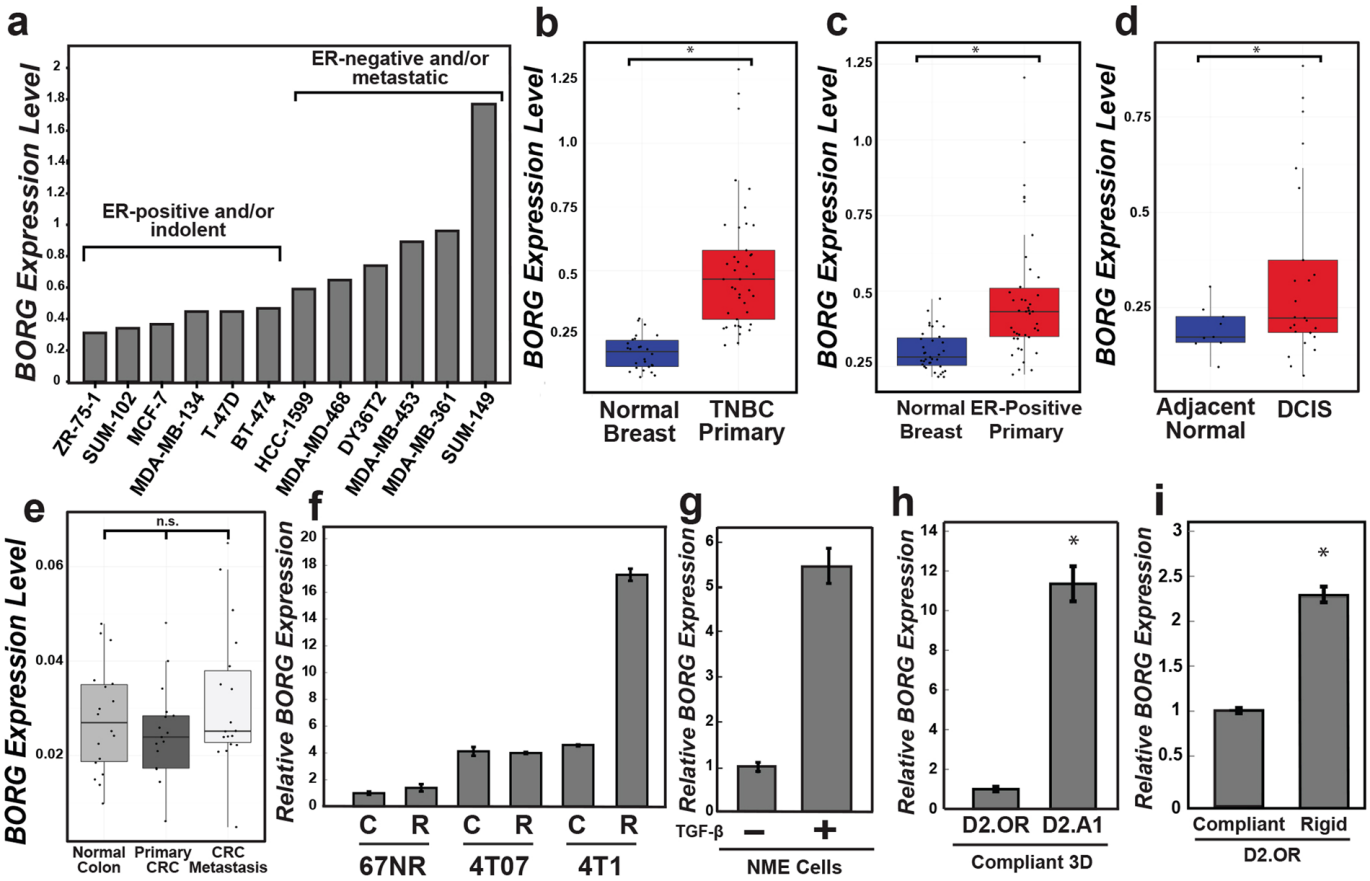
BORG expression correlates with the malignant and metastatic potential of breast cancers. (**a**) BORG expression across common breast cancer cell lines as determined by publicly available RNA-seq datasets. (**b** and **c**) BORG expression in 42 primary TNBC (**b**) or 42 ER-positive (**c**) breast tumors as compared to corresponding normal tissue using publicly available RNA-seq datasets (*P < 0.01; Mann-Whitney *U* Test). (**d**) BORG expression in 25 ductal carcinoma *in situ* (DCIS) lesions and 10 normal breast tissue organoids (*i.e*. DCIS; *P < 0.01; Mann-Whitney *U* Test). (**e**) BORG expression in normal colonic, primary colorectal, and colorectal metastasis tissue derived from 18 patients and analyzed using publicly available RNA-seq datasets (n.s.≈ *P* = 0.4622; Mann-Whitney *U* Test). (**f**) BORG expression was quantified *via* qRT-PCR in the 4T1 progression series propagated in compliant [C] or rigid [R; 3 mg/ml type I collagen] 3D-cultures. (**g**) TGF-β1 (5 ng/ml) stimulated BORG expression in NME cells undergoing EMT. (**h**) BORG expression was significantly higher in metastatic D2.A1 cells *versus* their latent D2.OR counterparts propagated in 3D-culture (*P < 0.05). (**i**) Rigid 3D-cultures significantly induced BORG expression in D2.OR organoids (*P < 0.05).

Included amongst the 17 differentially expressed lncRNAs identified *via* this method was BORG, which is an intergenic lncRNA approximately 2.8 kb in length that is essential in driving cell survival in response to a wide variety of cellular stress insults (*e.g*., heat shock, hypoxia, and nutrient deprivation^14^; Valadkhan *et al*., *unpublished data*). Disseminated tumor cells also face immense metabolic, oxidative, and physical stresses during transit to and within the metastatic microenvironment. As such, we inferred that BORG may promote the metastatic progression of breast cancer cells by fostering their survival as they traverse the metastatic cascade. Accordingly, a cohort of common human breast cancer cell lines showed increased levels of BORG expression in aggressive, largely ER-negative cell lines *versus* their indolent and predominantly ER-positive counterparts (Fig. 1a). More importantly, primary tissue samples derived from TNBC patients expressed significantly greater quantities of BORG as compared to normal, non-malignant mammary tissues (Fig. 1b). Elevated BORG expression was also observed in human patients with ER-positive tumors (Fig. 1c), and in human biopsies of non-invasive ductal carcinoma *in situ* lesions (Fig. 1d). In stark contrast, BORG expression was unchanged when comparing normal colonic epithelium, primary colorectal cancer (CRC), and even CRC metastases^23^ (Fig. 1e), signifying that the oncogenic activities of BORG transpire in a cell- and tissue-specific manner. Collectively, these findings indicate that aberrant BORG expression strongly correlates with the transformation and development of mammary carcinomas, as well as with their metastatic progression.

Historically, the functions of BORG have been discerned in various murine cell-based systems (*e.g*., myoblasts, bone marrow stromal cells, mammary epithelial cells)^14,24^, which prompted us to initially investigate the potential associations of BORG with breast cancer development and metastasis across several murine breast cancer models. In doing so, we quantified BORG expression across the murine 4T1 progression series^25^, which is a model of TNBC comprised of the nonmalignant 67NR, systemically invasive but non-metastatic 4T07, and the highly metastatic 4T1 cell lines. We also propagated these 4T1 derivatives in compliant and rigid (*e.g*., 3 mg/ml Type I collagen) 3D-cultures as a means to approximate the mechanical rigidity encountered by breast cancer cells in metastatic and primary tumor sites^26,27^. Figure 1f shows that BORG expression directly correlated with the extent of microenvironmental rigidity, and with the aggressiveness of each individual 4T1 derivative. Additionally, BORG expression was induced robustly by TGF-β in NME cells (*i.e*., NMuMG cells transformed by EGFR overexpression^28^) as they traversed the EMT program and thereby acquire metastatic competency (Fig. 1g). Taken together, these findings suggest that the inappropriate upregulation of BORG expression is a common feature for the conversion of minimally or non-transformed mammary epithelial cells into mesenchymal and aggressive metastatic variants.

To effectively model metastatic latency and disease recurrence in breast cancer, we utilized the D2.HAN series which consists of various clonally related cell lines derived from the same premalignant murine hyperplastic alveolar nodule^29,30^. Although these cell lines exhibit similar malignant properties *in vitro*, they differ greatly in their metastatic competency in both experimental and spontaneous metastasis models. Indeed, D2.OR cells are incapable of forming metastases despite colonizing distant tissues (*i.e*.exhibit metastatic latency *in vivo*), while D2.A1 cells are highly metastatic in all experimental settings^30–33^. Similarly, D2.OR cells arrest when propagated in compliant 3D-cultures that recapitulate the microenvironment of the lung, whereas D2.A1 cells are highly proliferative when propagated under identical 3D-culture conditions^33^. Consistent with their proliferative and metastatic behaviors, we observed metastatic D2.A1 cells to express significantly higher levels of BORG relative to their nonmetastatic D2.OR counterparts (Fig. 1h). Moreover, when D2.OR cells were grown in rigid 3D-cultures, a microenvironment shown to enhance their proliferative and metastatic capabilities^26,33^, BORG expression levels were increased significantly (Fig. 1i). Collectively, these findings suggest that low BORG expression correlates with the maintenance of a non-proliferative, indolent state, while high expression of the lncRNA associates with metastatic phenotypes.

### BORG induces the reactivation of proliferative programs and outgrowth of latent D2.OR cells both *in vitro* and *in vivo*

**Figure 2:**
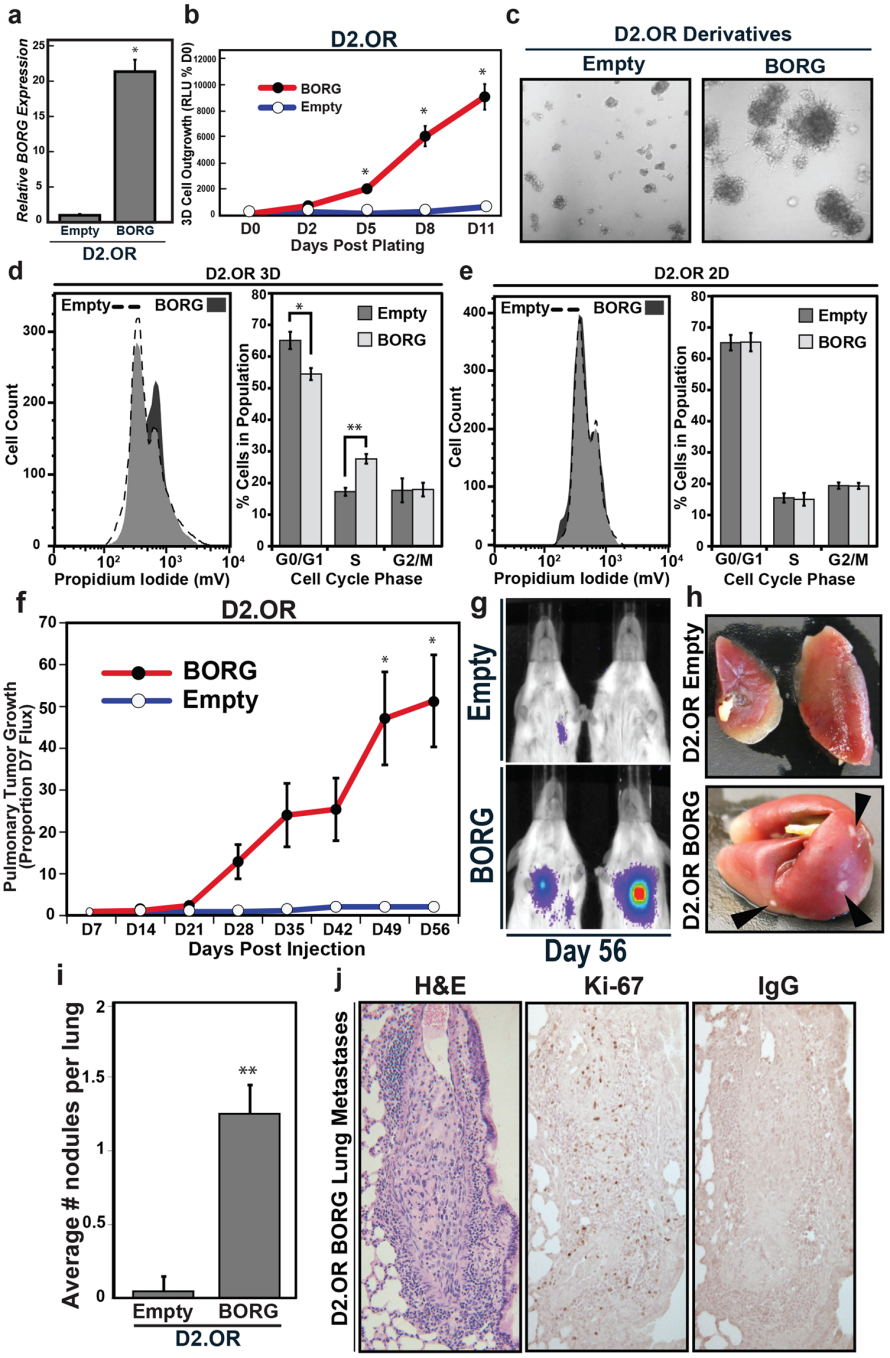
BORG induces the reactivation of proliferative programs and outgrowth of latent D2.OR cells both *in vitro* and *in vivo*. (**a**–**c**) Constitutive BORG expression (**a**) dramatically induced D2.OR organoid growth in 3D-cultures as determined by longitudinal bioluminescent assays (**b**; mean ± SEM; *P < 0.05) and phase contrast microscopy (**c**; 100X). (**d** and **e**) BORG promoted cell cycle progression in D2.OR cells propagated in 3D-cultures (**d**; mean ± SEM; *P < 0.05; **P < 0.01), but not in 2D-cultures (**e**). Grey shading indicates overlapping cell cycle distribution. (**f** and **g**) Quantification (**f**) and representative bioluminescent images (**g**) of parental (empty vector) or BORG-expressing D2.OR cells in the lungs of BALB/c mice (mean ± SEM; *P < 0.05). (**h** and **i**) Representative images (**h**) and quantification (**i**) of lungs and surface metastases produced by parental and BORG-expressing D2.OR cells from Panels h-j (mean ± SEM; **P < 0.01). (**j**) H&E, Ki-67, or isotype IgG control-stained tumor sections of lungs of BALB/c mice inoculated with BORG-expressing D2.OR cells.

To determine whether aberrant BORG expression simply associates with malignant and metastatic features, or whether this lncRNA acts to drive the reactivation of proliferative programs in latent D2.OR cells within metastatic settings, we stably overexpressed BORG in D2.OR cells to levels comparable with those observed in D2.A1 cells (Fig. 2a) and subsequently monitored differences in 3D-outgrowth by bioluminescence. As shown in Fig. 2b, BORG-expressing D2.OR cells formed significantly larger organoids over time as determined by quantification of bioluminescent output (Fig. 2b), and by phase contrast microscopy (Fig. 2c). Accordingly, we observed a greater proportion of BORG-expressing D2.OR cells to reside in the S phase of the cell cycle as compared to their parental counterparts, which were disproportionately arrested in G0/G1 phases (Fig. 2d). Interestingly, this BORG-dependent stimulation of cell cycle progression was not evident in traditional 2D-culture systems (Fig. 2e), implying that the proliferative effects of BORG are in part dependent upon the microenvironment. We similarly expressed BORG in D2.OR cells in a doxycycline-dependent manner (Supplementary Fig. 1a) and propagated these cells for 4 days in 3D-cultures to induce their proliferative arrest, at which point they were cultured in the absence or presence (1 μg/ml) of doxycycline for 5 additional days. Similar to its constitutive expression in D2.OR cells, inducible expression of BORG also significantly enhanced the 3D-outgrowth of D2.OR cells by day 9 as compared to empty vector controls (Supplementary Fig. 1b). As such, these results imply that BORG confers both an immediate proliferative advantage to D2.OR cells, as well as stimulates their malignant outgrowth after a period of non-proliferative latency has been previously established.

We next determined whether BORG-mediated reactivation of proliferative programs also occurred *in vivo*. Indeed, inoculating the lateral tail vein of BALB/c mice with BORG-expressing D2.OR cells resulted in the efficient metastatic colonization of lungs, a behavior that was entirely absent in parental D2.OR cells (Fig. 2f,g). Accordingly, macroscopic metastatic nodules were clearly more abundant in lungs harvested from mice injected with BORG-expressing D2.OR cells (Fig. 2h,i), and *ex vivo* culturing of these cells demonstrated that they retained aberrantly high levels of BORG expression and an increased capacity to grow in 3D-cultures (Supplementary Fig. 1c,d). Additionally, immunohistochemistry (IHC) analyses revealed multiple metastases that displayed dysplastic, irregular nuclei in lesions arising from BORG-expressing D2.OR cells, including numerous cells within these metastatic lesions that stained robustly for the cell proliferation marker Ki-67 (Fig. 2j). Collectively these findings establish that BORG acts as a mitogenic cue capable of inducing metastatic traits in non-metastatic breast cancer cells both *in vitro* and *in vivo*.

### Suppression of BORG inhibits outgrowth of metastatic D2.A1 cells both *in vitro* and *in vivo*

**Figure 3:**
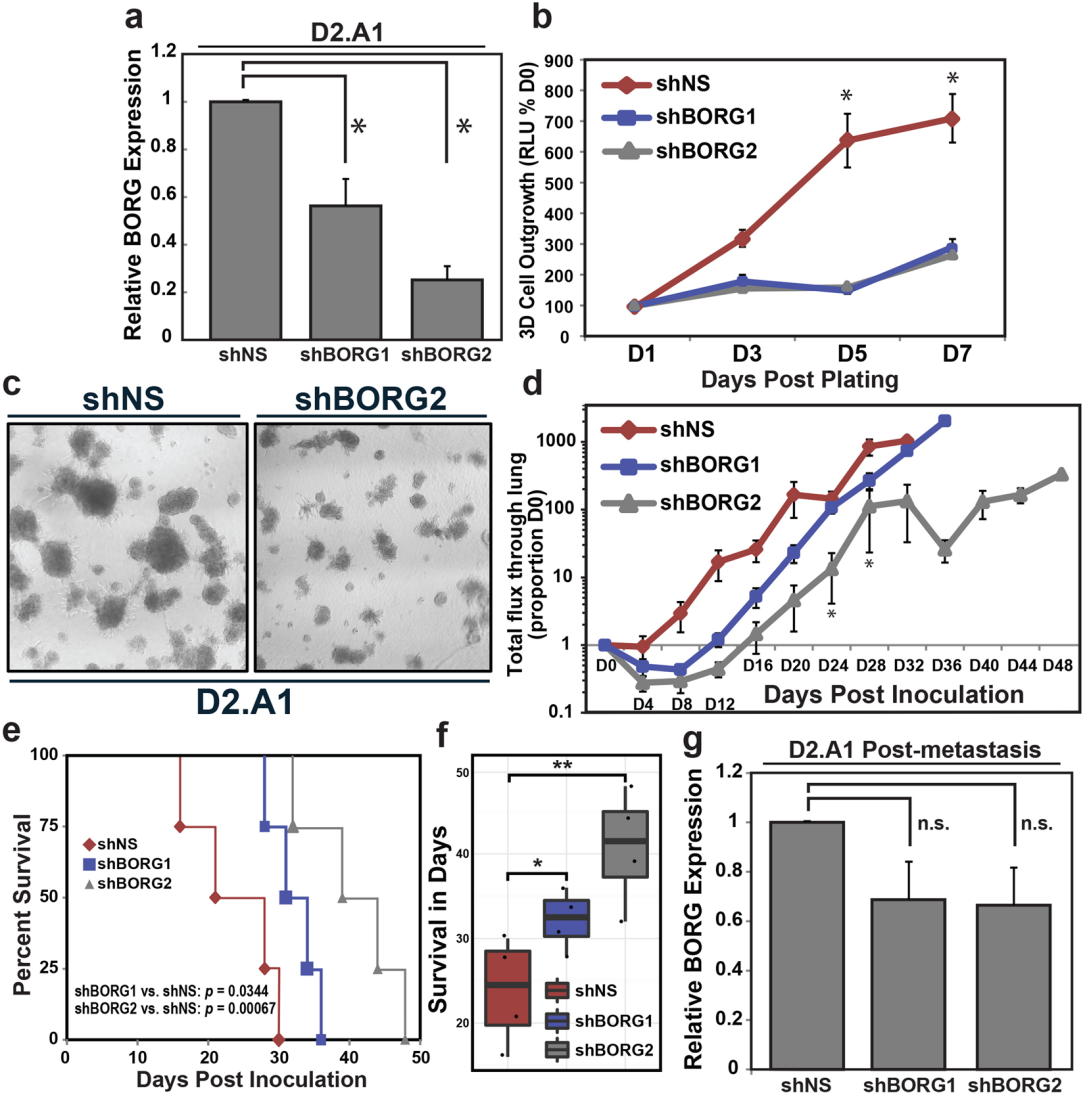
BORG-deficiency inhibits the growth of metastatic D2.A1 cells. (**a**) qRT-PCR analysis of BORG expression in D2.A1 cells engineered to express either of two independent shRNAs against BORG. (**b**) shRNA-mediated knockdown of BORG inhibited growth of D2.A1 cells in 3D-cultures, as quantified by bioluminescent readings. Data are the mean (±SEM; *P < 0.05). (**c**) Representative photomicrographs of organoids formed by D2.A1 derivatives taken 5 days post plating. (**d**) shBORG2 significantly impaired the metastatic activity of D2.A1 cells in the lungs of BALB/c mice (mean ± SEM; *P < 0.05). (**e** and **f**) BORG-deficiency positively impacted the overall survival of mice inoculated with D2.A1 cells (log-rank test *p* values annotated (**e**); *P < 0.05; **P < 0.0001 (**f**)). (**g**) BORG expression levels are elevated in the resultant D2.A1 populations after their isolation from lung metastases and subsequent *ex vivo* culture (mean ± SEM; n.s. ≈ not significant). Notably, BORG expression levels in the *ex vivo* culture of D2.A1 shBORG2 metastases were significantly higher than expression levels in pre-inoculated D2.A1 shBORG2 cells (**a**).

Although malignant human and murine breast cancer tissues demonstrate aberrant expression of BORG, the necessity of BORG to the development of their malignant features remains less clear. To address this question, we rendered metastatic D2.A1 cells deficient in BORG expression *via* shRNA-mediated knockdown (Fig. 3a) and subsequently seeded them into longitudinal 3D-culture growth assays. Inhibiting BORG expression using 2 distinct shRNAs against BORG dramatically inhibited the outgrowth of D2.A1 cells in 3D-cultures (Fig. 3b,c). Similarly, BORG-depleted D2.A1 cells were inoculated into the lateral tail vein of BALB/c mice and metastatic colonization of pulmonary tissues was monitored *via* bioluminescent output. Depletion of BORG significantly inhibited and delayed the formation of pulmonary metastases (Fig. 3d), while simultaneously prolonging the overall survival of these mice (Fig. 3e,f) in a manner that correlated with the efficiency of BORG knockdown (Fig. 3a). Although inhibition of BORG expression did not wholly prevent the metastatic outgrowth of D2.A1 cells, it should be noted that the metastases that arose had in fact lost expression of the shRNA against BORG, as evidenced by *ex vivo* cultures of D2.A1 lung metastases that no longer harbored significantly reduced levels of BORG compared to parental D2.A1 cells (Fig. 3g). This result implies that D2.A1 cells that possessed low levels of BORG were negatively selected during the development and outgrowth of D2.A1 tumors in the lungs of mice. Collectively, these findings indicate that the metastatic features of D2.A1 cells rely in part on their enhanced expression of BORG.

### BORG interacts physically with TRIM28 to promote metastatic phenotypes

**Figure 4:**
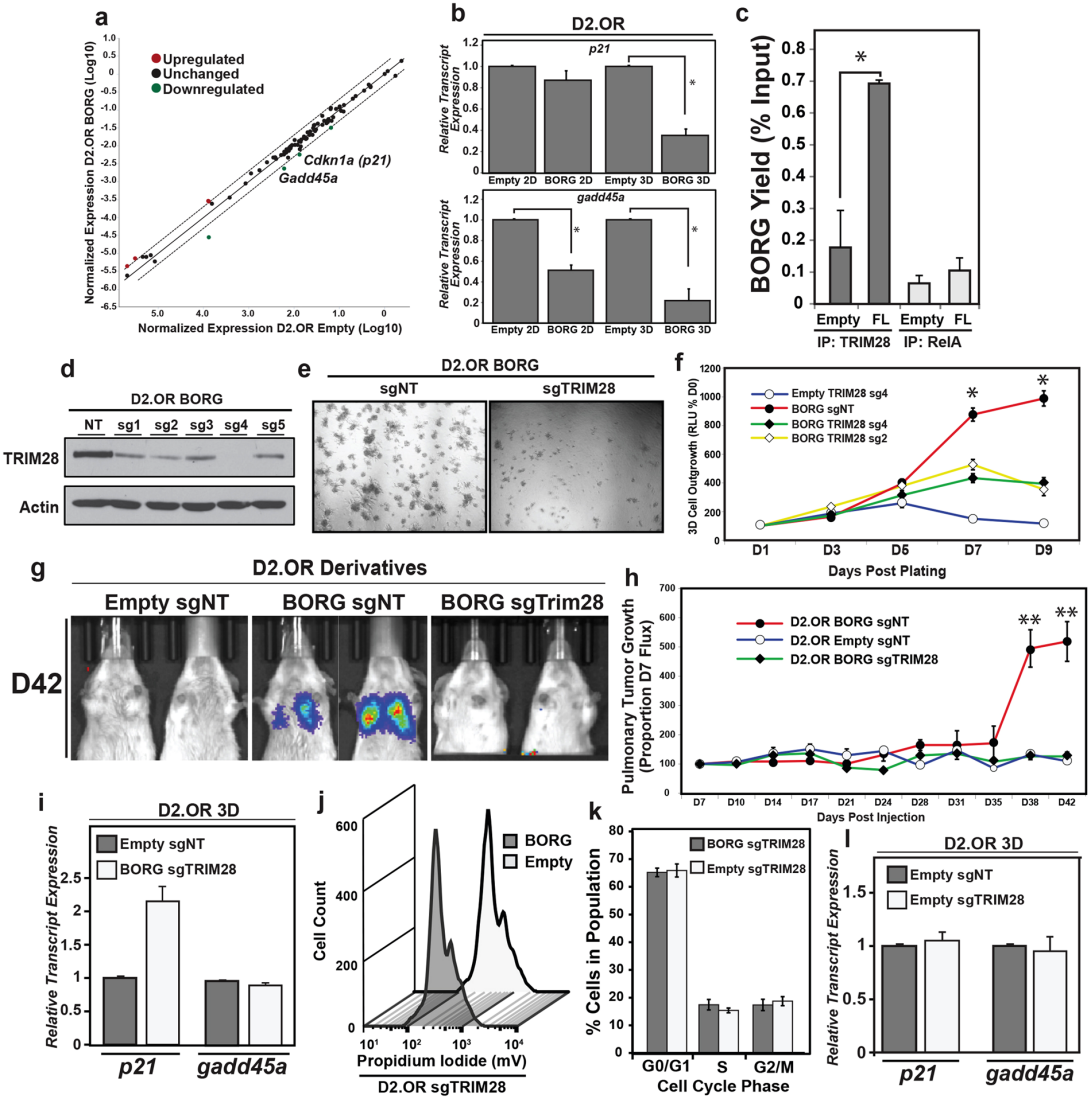
BORG interacts physically with TRIM28 to promote metastatic phenotypes. (**a**) The expression of 84 senescence-associated genes were compared between parental (empty) and BORG-expressing D2.OR organoids propagated for 7 days in 3D-cultures. Dashed lines represent 2-fold increase or decrease in transcript level. (**b**) BORG suppressed the expression of *cdkn1a (p21)* and *gadd45a* in D2.OR cells (mean ± SEM; *P < 0.05). (**c**) RNA-immunoprecipitation assay validated the specific interaction of TRIM28 with BORG. Immunoprecipitation of RelA served as negative control (mean ± SEM; *P < 0.05). (**d**–**f**) Inactivating TRIM28 expression in BORG-expressing D2.OR cells (**d**) significantly impaired their growth in 3D-cultures as determined by phase-contrast microscopy (**e**; 100X) and longitudinal bioluminescent assays (**f**; mean ± SEM; *P < 0.05). (**g** and **h**) Inactivating TRIM28 expression in BORG-expressing D2.OR cells prevented their outgrowth in the lungs of BALB/c mice (mean ± SEM; *P < 0.05). (**i**–**l**) TRIM28-deficiency in BORG-expressing D2.OR cells restored latency-associated expression levels of *p21* and *gadd45a* transcripts (**i**) and inhibited cell cycle progression (**j**). Inactivating TRIM28 expression in parental D2.OR cells failed to impact their cell cycle dynamics (**j** and **k)** or the abundance of *p21* and *gadd45a* transcripts (**l**). Refer to Supplementary Figure 6 for full-length, uncropped blot.

The molecular mechanisms underlying the pathological and proliferative activities of BORG remain unknown. Nonetheless, lncRNAs frequently exert their physiologic functions by modulating the expression of genes on a broad, genome-wide scale^34^. To decipher potential transcriptional alterations that drive the proliferative advantage afforded by BORG, we performed a targeted transcriptional array on parental and BORG-expressing D2.OR cells grown in 3D-culture to quantify the expression of transcripts known to influence senescence-associated pathways, and consequently, metastatic latency (Fig. 4a). Aberrant expression of select mediators of cell cycle progression was detected in BORG-expressing D2.OR cells, including a downregulation of the known tumor suppressors and cell cycle inhibitors, *cdkn1a (p21)* and *gadd45a* ^35,36^ (Fig. 4b).

Although lncRNAs employ a variety of mechanisms to alter gene expression, they frequently rely upon the formation of protein:lncRNA complexes capable of eliciting widespread transcriptional control throughout the genome^9,37–39^. To identify protein binding partners of BORG, we created biotinylated oligonucleotides complementary to BORG that were used to capture BORG-interacting proteins, which were subsequently identified by mass spectrometry analysis (Supplementary Fig. 2a,b; Valadkhan *et al.unpublished data*). Amongst the 12 proteins determined to interact directly with BORG was the E3 SUMO ligase TRIM28 (KAP1; Fig. 4c), a molecule previously speculated to function as an RNA-binding protein^40^. More recent evidence suggests that TRIM28 plays an oncogenic role in numerous human malignancies^41–44^, including several studies linking TRIM28 to the metastatic progression of breast cancers^17,45–47^. Interestingly, the ability of TRIM28 to enhance the malignancy of breast cancer cells transpires in part through its transcriptional repression of both *p21* and *gadd45a* ^15,16,18^, two transcripts whose expression is negatively regulated by BORG in D2.OR cells (Fig. 4b).

In light of the fact that TRIM28 interacts with BORG (Fig. 4c) and that aberrant BORG expression significantly downregulates two critical tumor suppressor genes whose expression is known to be modulated by TRIM28 (Fig. 4b), we postulated that the formation of BORG:TRIM28 complexes would underlie the oncogenic activities of BORG. As such, we knocked out TRIM28 in parental and BORG-expressing D2.OR cells using the CRISPR/Cas9 genome editing approach in a highly targeted manner (Fig. 4d), resulting in the generation of an inappropriate stop codon elicited by a single nucleotide insertion and frameshift in the second exon of BORG (Supplementary Fig. 2c,d). When propagated in 3D-cultures, TRIM28-deficient, BORG-expressing D2.OR cells formed dramatically smaller organoids (Fig. 4e) and exhibited significantly reduced longitudinal outgrowth (Fig. 4f) as compared to their TRIM28-proficient counterparts. Notably, this pattern of 3D-outgrowth transpired irrespective of the specific single guide RNA (sgRNA) used to target TRIM28 (Fig. 4f), indicating that these growth-inhibitory effects were not due to off-target activities of the guide RNA. More importantly, abrogating TRIM28 expression in BORG-expressing D2.OR cells also abolished their metastatic competency when inoculated intravenously into BALB/c mice (Fig. 4g,h). Quantification of *gadd45a* and *p21* levels in these BORG/sgTRIM28-expressing D2.OR cells revealed that TRIM28 was indeed essential for the BORG-mediated downregulation of these senescence-associated cell cycle inhibitors (Fig. 4i). Accordingly, cell cycle analyses showed that BORG/sgTRIM28-expressing D2.OR cells displayed an (i) increased frequency of cells arrested in G0/G1 phase, and (ii) decreased frequency of cells in S phase as compared to BORG-expressing cells grown in 3D culture (Figs. 4j,k, 2d). Interestingly, silencing of TRIM28 failed to alter the cell cycle dynamics (Fig. 4j,k) and expression of *gadd45a* and *p21* in parental D2.OR cells (Fig. 4l). Taken together, these findings suggest that BORG confers unique regulatory features to TRIM28.

**Figure 5:**
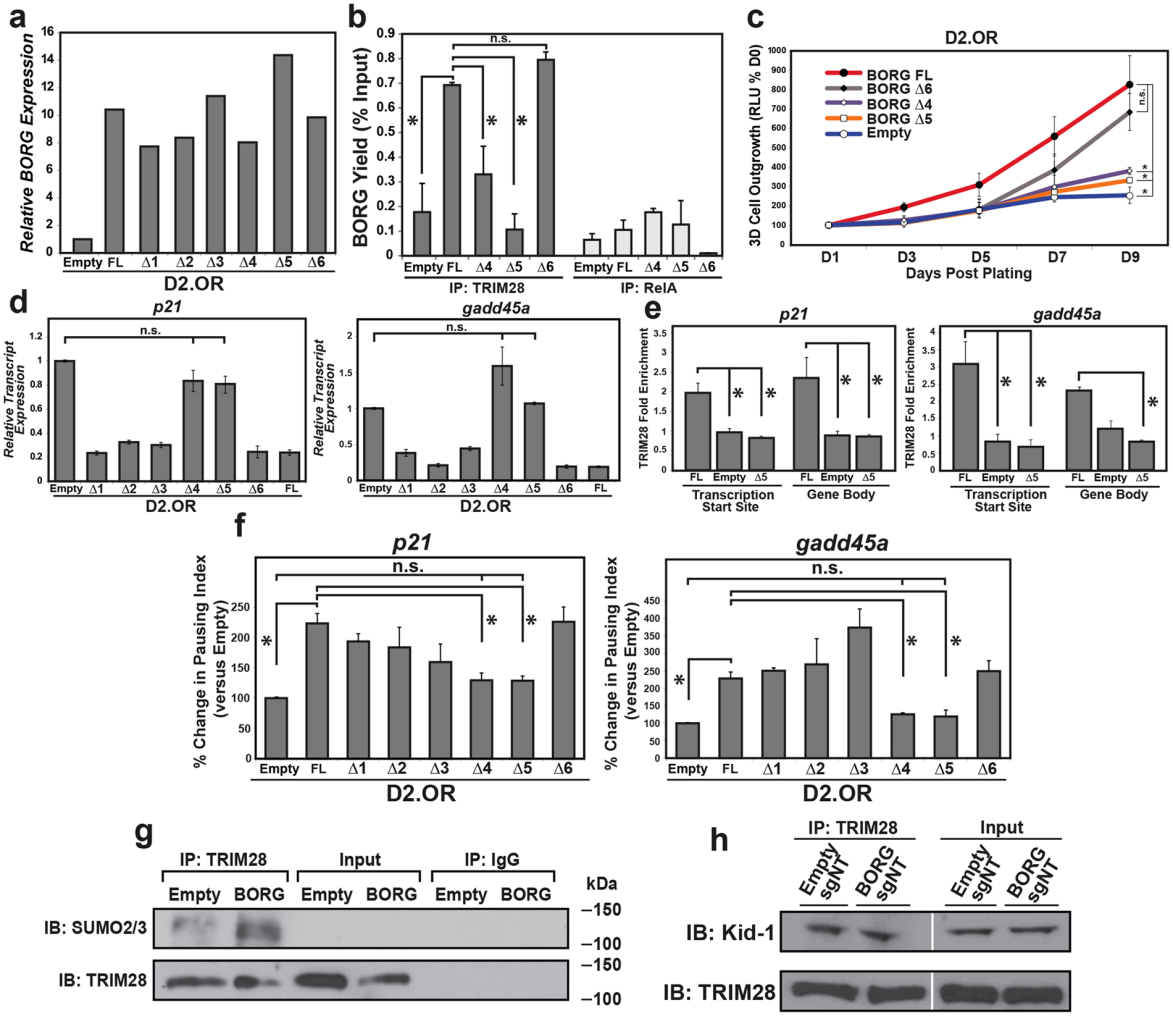
BORG localizes and enhances the repressive effect of TRIM28 at multiple genomic loci. (**a**) Expression analyses of full-length (FL) BORG and its internal deletion mutants (Δ1-6) in D2.OR cells. (**b**) RNA-immunoprecipitation analyses of TRIM28 immunocomplexes (RIP) demonstrated the necessity of BORG sequences encoded by deletions 4 (Δ4) and 5 (Δ5) in binding TRIM28. Identical IP of RelA served as a negative control. Data are the mean (±SEM; *P < 0.05; n.s. ≈ not significant). (**c**) Expression of BORG Δ4 and Δ5 mutants failed to stimulate the outgrowth of D2.OR organoids in 3D-culture. Data are the mean ( ± SEM; *P < 0.05). (**d**) Expression of BORG Δ4 and Δ5 mutants failed to repress *p21* and *gadd45a* transcript levels in D2.OR organoids. Data are the mean (±SEM; *P < 0.05). (**e**) TRIM28 ChIP-qPCR assays demonstrated the inability of BORG Δ5 mutant to localize TRIM28 to the transcription start sites (TSS) or gene bodies of the p21 and gadd45a loci. Data are the mean (±SEM; *P < 0.05). (**f**) Expression of BORG Δ4 and Δ5 mutants failed to elicit proximal promoter pausing at the p21 and gadd45a loci as determined by RNA Polymerase II (Pol II) ChIP analyses of D2.OR organoids. Data are the mean (±SEM; *P < 0.05). (**g**) BORG stimulated the sumoylation and activation of TRIM28 in D2.OR cells as determined by immunoblotting (IB) TRIM28 immunocomplexes (IP) with anti-SUMO2/3 antibodies. Refer to Supplementary Figure 6 for full-length, uncropped blot. (**h**) BORG expression failed to impact the binding of TRIM28 to the KRAB-ZNF protein, Kid1, in D2.OR cells as determined by immunoblotting (IB) TRIM28 immunocomplexes (IP) with anti-Kid1 antibodies. Refer to Supplementary Figure 6 for full-length, uncropped blot; white line represents merger of non-bordering lanes.

To further elucidate the interaction of BORG with TRIM28, we expressed BORG mutants that housed sequential internal deletions throughout the length of the transcript (Fig. 5a)^24^, and subsequently assessed the preservation of BORG:TRIM28 complexes *via* RNA-immunoprecipitation experiments. Indeed, quantifying the abundance of BORG captured by TRIM28 immunocomplexes identified 2 BORG mutants (deletions 4 & 5, referred to as Δ4-BORG and Δ5-BORG, respectively) that displayed significantly reduced capacity to bind TRIM28 (Fig. 5b), indicating that sequences within this ~1000 bp region of BORG were critical in binding TRIM28 (Supplementary Fig. 3a). Accordingly, D2.OR cells engineered to stably express Δ4-BORG and Δ5-BORG mutants lost their competency to proliferate in 3D-cultures (Fig. 5c; Supplementary Fig. 3b,c); they also re-expressed *p21* and *gadd45a* to levels comparable to those seen in parental D2.OR cells (Fig. 5d). Collectively, these results demonstrate that TRIM28 is imperative to BORG-mediated stimulation of proliferative and metastatic programs, doing so by repressing the expression of the tumor suppressor genes, *p21* and *gadd45a*.

### BORG localizes and enhances the repressive effect of TRIM28 at multiple genomic loci

Nuclearly localized lncRNAs such as BORG^24^ have been shown to regulate gene expression by localizing transcription factors to various genomic loci in order to enhance their gene-modulatory behavior^38,39^. The BORG binding partner TRIM28 localizes to the nucleus where it has been shown to interact with the promoters of genomic loci *via* its consensus DNA-binding motif to inhibit transcription through regulation of RNA Polymerase II (Pol II) pause and release^48,49^. Interestingly, the loci for *p21* and *gadd45a* both possess TRIM28 DNA consensus binding sites located 25 and 103 base pairs, respectively, downstream of the transcription start site (Supplementary Fig. 3d). To determine whether BORG modulates the capacity of TRIM28 to interact at these loci, we performed chromatin immunoprecipitation (ChIP) assays followed by qRT-PCR on parental and BORG-expressing D2.OR cells grown in 3D-culture. In doing so, we determined that BORG enriched the binding of TRIM28 to both the proximal promoters and gene bodies of the *p21* and *gadd45a* loci (Fig. 5e). Importantly, no such enrichment was evident in D2.OR cells that expressed the Δ5-BORG mutants (Fig. 5e), suggesting that the binding of BORG to TRIM28 serves as an essential step in localizing this E3 SUMO ligase to various genomic loci. Similar ChIP experiments revealed that full-length BORG, but not its Δ4-BORG and Δ5-BORG mutants, significantly increased the pausing index of Pol II at both of these loci when compared to parental D2.OR cells (Fig. 5f; *i.e*., the ratio of Pol II occupancy at the transcription start site *versus* its occupancy at the gene body^48^). Correspondingly, only D2.OR cells that expressed Δ4-BORG and Δ5-BORG mutants showed a significant reduction in the pausing index at these loci when compared to full-length BORG (Fig. 5f). Additionally, we observed that the binding of BORG to TRIM28 induces its auto-sumoylation activity (Fig. 5g), a feature that correlates with the transcriptional repressive activity of TRIM28^50,51^. It is important to note that heterologous expression of BORG failed to enhance the interaction of TRIM28 with Kid-1 (Fig. 5h), a KRAB-ZNF commonly found within TRIM28 transcriptional co-repressor complexes^52^. Taken together, these findings highlight the specificity of BORG in localizing and enhancing TRIM28-mediated transcriptional repression at unique genomic loci, events that transpire independent of its co-repressor complex.

### BORG promotes the activation of survival and metastasis pathways in D2.OR cells

**Figure 6:**
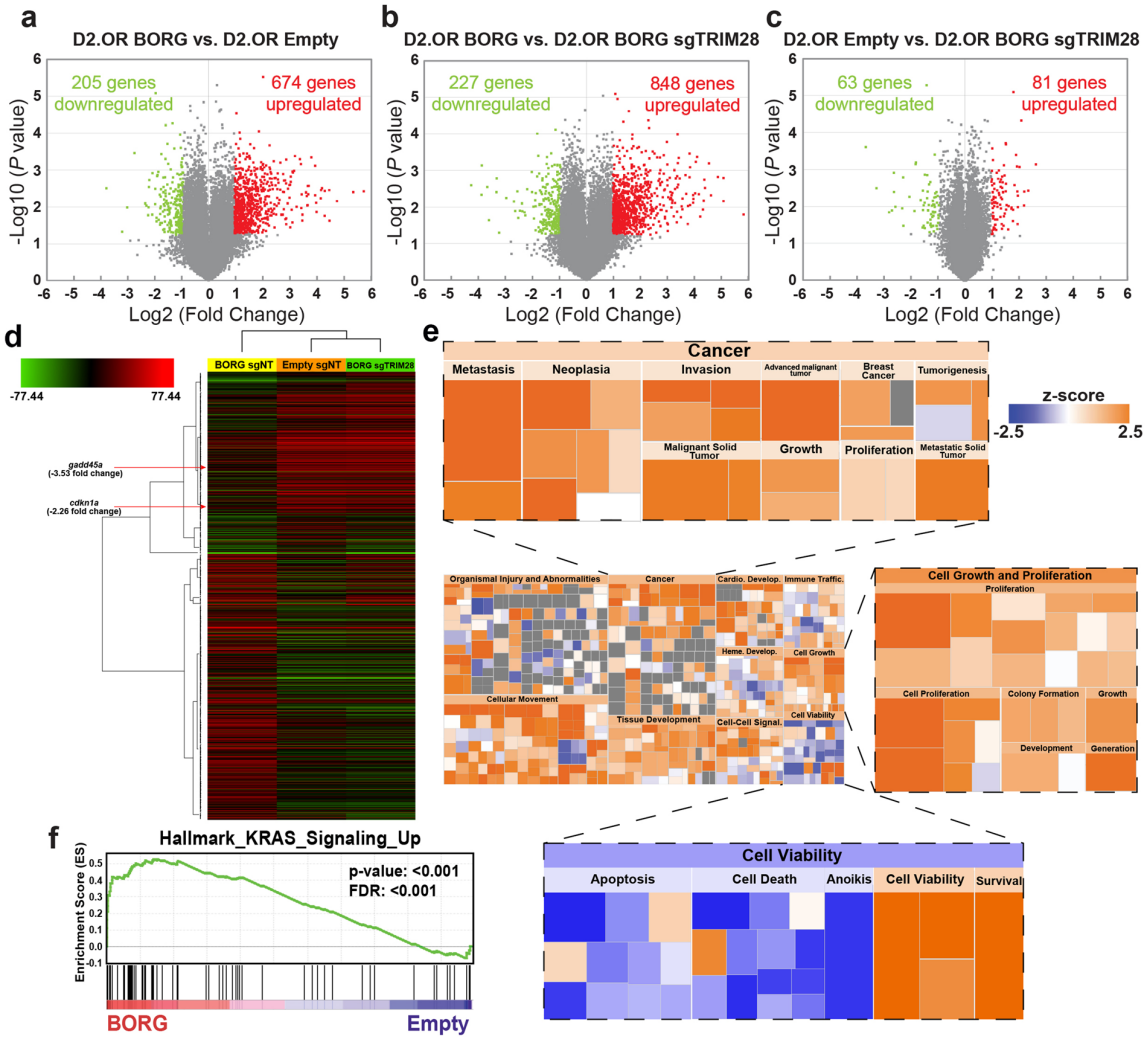
BORG confers a unique genome-wide transcriptional signature to latent D2.OR cells. (**a**–**c**) Volcano plots showing differentially expressed transcripts in D2.OR-BORG *versus* parental D2.OR cells (**a**), D2.OR-BORG *versus* TRIM28-deficient D2.OR-BORG cells (**b**), and parental D2.OR *versus* TRIM28-deficient D2.OR-BORG cells (**c**) grown in 3D-culture for 7 days prior to Affymetrix GeneChip Mouse Gene 2.0 ST Array analysis. Cut-off criteria was an absolute change ≥ 2.0-fold (*P < 0.05). (**d**) Hierarchical clustering of transcriptional signatures arising from ~1150 genes whose expression was significantly altered in the three groups analyzed in Panels A–C. Fold difference in expression of *p21* and *gadd45a* in D2.OR-BORG cells versus parental D2.OR cells is annotated. (**e**) Hierarchical heatmap obtained using the Downstream Effects Analysis feature of IPA software to define BORG-regulated biological processes. Representative biological functions expanded in subpanels. Z-score represents a statistical measure of the activation state of each biological process (increased: orange; decreased: blue). (**f**) GSEA demonstrated enrichment of the hallmark KRAS gene signature in D2.OR-BORG cells *versus* their parental counterparts.

The genome-wide promiscuity of TRIM28, as well as the ubiquitous nature of lncRNAs to comprehensively alter cellular transcriptional networks led us to hypothesize that BORG:TRIM28 complexes are capable of compelling metastasis by inducing global genomic reprogramming beyond that of *p21* and *gadd45a*. Accordingly, we propagated parental, BORG-, and BORG/sgTRIM28-expressing D2.OR cells in 3D-culture for 7 days, at which point total RNA was isolated and subsequently subjected to Affymetrix GeneChip Mouse Gene 2.0 ST Array analysis to assess genome-wide transcriptomic profiles governed by BORG in a TRIM28-dependent and -independent manner. In doing so, we identified 674 genes whose expression was significantly upregulated by BORG in D2.OR cells, as well as an additional 205 genes whose expression was significantly downregulated (Fig. 6a; ±2-fold cut-off; *P* < 0.05). Moreover, rendering BORG-expressing D2.OR cells deficient in TRIM28 expression resulted in 848 genes being significantly upregulated, with another 227 being significantly downregulated (Fig. 6b). Interestingly, compared to parental D2.OR cells, only a modest number of genes (*e.g*., 81 upregulated an 63 downregulated) were significantly impacted by deleting TRIM28 expression in BORG-expressing D2.OR cells (Fig. 6c), implying that (i) the capacity of TRIM28 to regulate gene expression is greatly enhanced by its binding to BORG, and (ii) the tumor promoting properties of BORG similarly require TRIM28 to exert widespread transcriptional alterations operant in driving non-metastatic breast cancer cells to activate proliferative programs. Accordingly, hierarchical clustering of ~1150 genes whose expression was significantly altered in the three experimental groups revealed that BORG/sgTRIM28 and parental D2.OR cells maintained transcriptional signatures that were highly analogous to one another, with both groups differing substantially from their BORG-expressing counterparts (Fig. 6d). Collectively, these analyses indicate that the pro-metastatic effects of BORG predominantly reflect its ability to bind and activate TRIM28, thereby altering genome-wide transcript expression.

To further analyze this unique BORG-dependent transcriptional profile, we utilized Ingenuity Pathway Analysis (IPA) to characterize the cellular processes and functions influenced by BORG in D2.OR cells. As expected, BORG-expressing D2.OR cells showed broad activation of pathways associated with metastasis and cell proliferation (Fig. 6e), including extensive enrichment of hallmark KRAS signaling (Fig. 6f), a pathway highly associated with proliferative phenotypes in human breast cancers^53^. Similarly, BORG-expressing D2.OR cells demonstrated enrichment of EMT signatures, as well as transcriptional signatures associated with aggressive, basal-like breast cancers *versus* their more indolent luminal counterparts (Supplementary Fig. 4a,b). Equally important, these analyses also identified a significant decrease in *gadd45a-* and *p21-*dependent enhancement of cell cycle progression solely in BORG-expressing D2.OR cells (Supplementary Fig. 4c), a finding that correlates with the ability of BORG:TRIM28 complexes to downregulate the expression of these transcripts (Figs 4b, 5d). Taken together, these findings indicate that BORG enhances genome-wide flux through proliferative pathways that are associated with malignant transformation and the acquisition of aggressive pathoclinical features of breast cancer.

**Figure 7:**
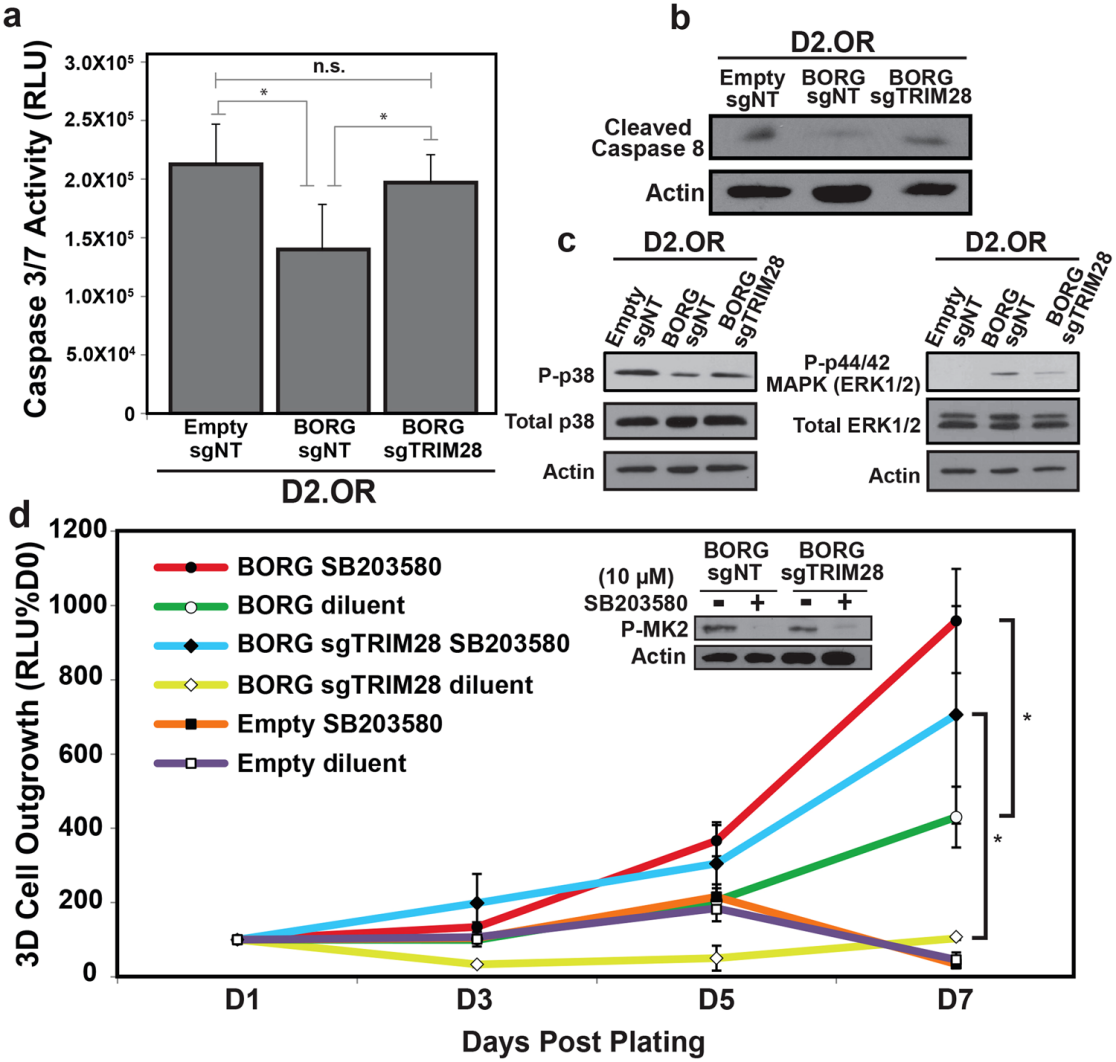
BORG expression modifies metastasis-associated pathways to enhance cellular survival and outgrowth. (**a**) BORG protects D2.OR cells from anoikis in a TRIM28-dependent manner as determined by Caspase-Glo 3/7 assays. Data are mean (±SEM; *P < 0.05). (**b**) BORG promotes the survival of D2.OR organoids in a TRIM28-dependent manner as determined by immunoblotting for cleavage of Caspase 8. Refer to Supplementary Figure 6 for full-length, uncropped blot. (**c**) BORG relies on TRIM28 to enhance the phosphorylation and activation of ERK1/2 and reduce that of p38 MAPK in D2.OR derivatives grown in 3D-culture for 7 days. Refer to Supplementary Figure 6 for full-length, uncropped blot. P ≈ phosphorylated. (**d**) p38 MAPK inhibition (SB203580; 10 μM) enhances the 3D-outgrowth of BORG-expressing D2.OR derivatives. Data are mean (±SEM; *P < 0.05). Immunoblot analysis depicts the phosphorylation and activation of MK2, a downstream target of p38 MAPK, in D2.OR derivatives treated with or without SB203580 for 5 days in 3D-culture. Refer to Supplementary Figure 6 for full-length, uncropped blot.

In addition to identifying proliferative pathways targeted by BORG in non-metastatic D2.OR cells, our cellular network analyses also demonstrated extensive upregulation of pro-survival pathways, as well as widespread downregulation of cell death pathways when BORG was overexpressed in D2.OR cells (Fig. 6e). To better understand the cellular effects downstream of this transcriptional pattern, we subjected D2.OR variants to various forms of stress, including anoikis, a condition commonly encountered by disseminating cells as they traverse the metastatic cascade^54^. In doing so, D2.OR derivatives were cultured in poly-HEMA coated tissue culture plates prior to monitoring differences in Caspase 3 and 7 activity as a surrogate for cell survival. Figure 7a shows that BORG-expressing D2.OR cells contained significantly lower levels of Caspase 3 and 7 activity, indicating that they were more resistant to anoikis as compared to their parental counterparts. Similarly, the extent of caspase 8 cleavage was dramatically reduced in BORG-expressing D2.OR cells subjected to extended 3D-culture (*i.e*.,12 days) relative to those measured in parental and BORG/sgTRIM28 cells (Fig. 7b). As such, these data provide strong evidence that BORG:TRIM28 complexes confer persistent survival advantages to D2.OR cells propagated in microenvironments that recapitulate the pulmonary metastatic niche.

Recent evidence suggests that disseminated cells rely heavily upon the activation of p38 MAPK signaling to enhance their survival when confronted with noxious and stressful conditions, a survival response that is frequently coupled to the inactivation of ERK1/2 signaling^3,55^. The appearance of high p38 MAPK:ERK1/2 activity ratios thereby manifests as viable, nonproliferative states associated with metastatic latency^3,55^. Accordingly, we found that as BORG-expressing D2.OR cells acquired metastatic features, they readily suppressed p38 MAPK activity and stimulated that of ERK1/2 (Fig. 7c). Interestingly, this BORG-mediated switch in MAPK dependency was reliant upon TRIM28, as dormant BORG/sgTRIM28 D2.OR cells displayed high p38 MAPK:ERK1/2 activity ratios (Fig. 7c). Along these lines, pharmacological inhibition of p38 MAPK activity *via* treatment with SB203580 significantly enhanced the longitudinal 3D-outgrowth in BORG-expressing D2.OR cells, as well as in their TRIM28-deficient counterparts (Fig. 7d). It should be noted that treating parental D2.OR cells with SB203580 failed to impact their outgrowth proficiency in 3D-cultures (Fig. 7d), indicating the necessity of BORG to activate ERK1/2 and other ancillary pathways as a means to promote metastatic behavior. Taken together, these data imply that the pro-survival effects of BORG act independently of the p38 MAPK signaling axis, and that the induction of p-ERK1/2 levels by BORG:TRIM28 complexes (Fig. 7c) is critical to inducing a proliferative phenotype in latent D2.OR cells upon p38 inhibition.

### Malignant and metastatic human breast cancer tissues express aberrantly high levels of BORG

**Figure 8:**
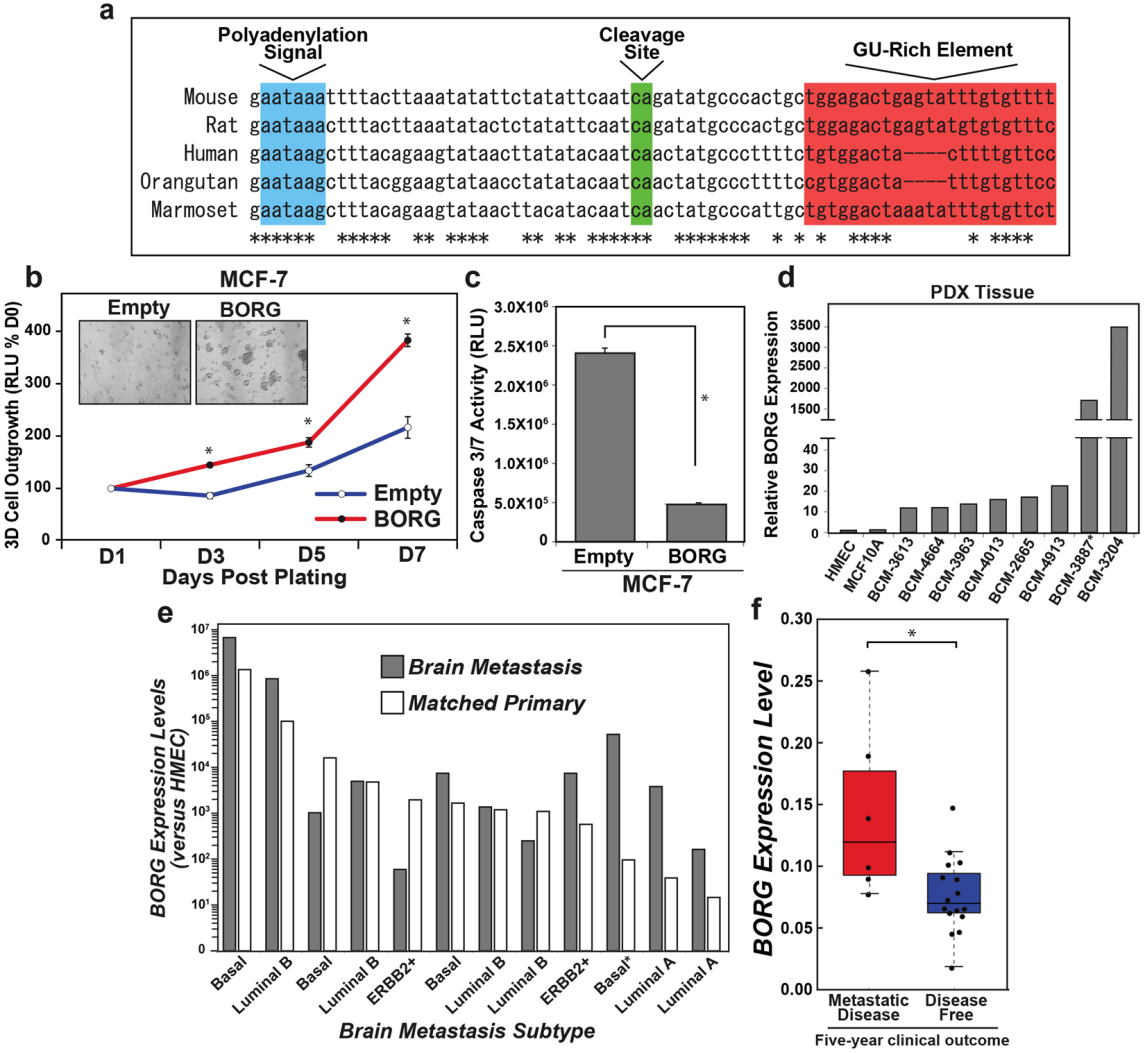
Malignant and metastatic human breast cancer tissues express aberrantly high levels of BORG. (**a**) Sequences governing functional 3′ end formation of BORG are conserved throughout multiple mammalian species. Depicted is the syntenic region corresponding to the 3' end of the mouse BORG gene (nucleotides 2601 to +39). Cleavage site marks end of BORG transcript. Asterisks denote conserved bases among all annotated species. (**b**) Murine BORG stimulated the longitudinal growth of human MCF-7 cells in 3D-cultures. Inset depicts representative photomicrographs of organoids 7 days post plating (100X; mean ± SEM; *P < 0.05). (**c**) Murine BORG protected human MCF-7 cells from anoikis as determined by Caspase-Glo 3/7 assays. Data are mean (±SEM; *P < 0.05) of two independent experiments performed in triplicate. (**d**) qRT-PCR analysis of BORG transcript abundance in patient-derived xenograft (PDX) tumor tissue compared to normal human mammary epithelial cells (HMEC) derived from reduction mammoplasty. *Denotes tissue derived from recurrent metastatic BC lesion. (**e**) qRT-PCR analysis of BORG transcript abundance in matched primary breast tumors and their corresponding brain metastases. BORG levels were normalized to HMECs. *Denotes tissue derived from recurrent metastatic breast cancer lesion. (**f**) Comparison of BORG expression in primary TNBC tumors obtained from patients who develop metastatic recurrence within 5 years of initial treatment *versus* the levels measured in patients who remain disease-free for >5 years (*P < 0.01; Mann-Whitney *U* Test).

Although mechanistic insights into the pathologic effects of BORG were garnered primarily from murine models that utilized the mouse-specific mature transcript of BORG, human BORG possesses a degree of conserved synteny and sequence homology that greatly mirrors that seen in the majority of identified lncRNAs^56^. Such conservation includes highly homologous genomic regions towards the distal end of BORG that dictate its functional 3’ end formation (*i.e*. poly-adenylation and transcript cleavage; Fig. 8a), as well as conserved domains throughout the third exon of murine BORG (Supplementary Fig. 5). Included in this region is a ~625 nucleotide stretch that exhibits ~63% sequence homology between human and murine BORG. Moreover, evolutionary studies of syntenic lncRNAs have revealed that the physiologic functions of such transcripts are strongly preserved between species, even among orthologous lncRNAs that exhibit extraordinarily low sequence conservation^56,57^. To determine whether human and murine BORG harbor functional conservation, we engineered human MCF-7 cells to express the murine ortholog of BORG and assessed for phenotypic alterations in their proliferation and survival. Interestingly, heterologous expression of murine BORG significantly enhanced the longitudinal outgrowth of MCF-7 cells in 3D-cultures (Fig. 8b), as well as reduced their sensitivity to anoikis (Fig. 8c). As such, these findings signify that the functional components of murine BORG are sufficiently conserved across species and remain competent in modifying similar pro-proliferative and pro-survival pathways in human breast cancer cells.

Based on the aforementioned findings, we speculated that the oncogenic activities of BORG are essential to the metastatic spread of disease in breast cancer patients. To test this supposition, we acquired multiple cohorts of human primary and metastatic breast cancer samples, including patient-derived xenograft (PDX) tissue established from patients harboring a wide range of pathoclinical features^58^ (Supplementary Table 1). These malignant tissues clearly expressed increased levels of BORG as compared to normal human mammary epithelial cells (HMECs), and to indolent MCF-10A cells (Fig. 8d). Notably, two samples possessed striking upregulation of BORG expression, including the PDX samples that (i) exhibited the greatest extent of metastasis (BCM-3204), and (ii) was derived from a recurrent, metastatic lesion (BCM-3887).

Additionally, we quantified BORG expression in a cohort of matched primary breast tumors and their corresponding CNS metastases. When compared to HMECs, both sources of malignant tissue exhibited exceptional escalation of BORG expression (Fig. 8e). Moreover, aberrant BORG expression trended to occur more readily in aggressive subtypes of primary tumors (*i.e*., Basal and Luminal B) that harbored higher quantities of BORG as compared to more indolent BC subtypes (*i.e*., Luminal A; Fig. 8e). Notably, the majority of brain metastases possessed amplified levels of BORG when compared to their matched primary tumor, while the metastatic sample exhibiting the most robust amplification of BORG as compared to its corresponding primary lesion was derived from a recurrent metastatic lesion (Fig. 8e). These findings therefore imply that aberrant BORG expression may compel the relapse of malignant disease in breast cancer patients in a manner analogous to its ability to drive latent D2.OR cells to attain metastatic competency both *in vitro* and *in vivo* (Fig. 2b,f). Along these lines, we quantified the levels of BORG expression in TNBC lesions that resulted in metastatic relapse within 5 years of initial patient diagnosis and treatment, which were subsequently compared to those measured in TNBC tumors obtained from patients who remained disease-free for 5 years post-treatment^22^. Importantly, Fig. 8f shows that BORG expression was indeed significantly higher in TNBC patients who rapidly developed metastatic recurrence (*i.e*., <5 years) as compared to patients who remained disease-free during this same timeframe. Collectively, these findings suggest that primary tumors that house robust expression of BORG shed malignant cells that are predisposed to establishing clinically relevant secondary lesions, particularly in TNBCs that currently lack effective prognostic and predictive biomarkers.

## Discussion

Current dogma states that the proficiency of circulating tumor cells to extravasate from the vasculature and successfully colonize metastatic niches dictates the relative competency of primary tumors to establish overt distant metastases^59^. This metastatic “bottleneck” is characterized by immense cellular stress at foreign sites of colonization, which often is sufficient to impede proliferation and trigger apoptosis in disseminated cells as they struggle to cope with the loss of junctional and adhesive signaling networks^54,60,61^. Consequently, micrometastatic lesions fail to propagate due to their inability to proliferate at rates that outpace their death by apoptosis. Our findings clearly indicate that BORG is uniquely poised to influence the metastatic spread of breast cancers through its ability to target both sides of this equation – *i.e*., to stimulate proliferative and survival signaling systems.

The oncogenic activities of BORG are mediated at a genome-wide level by interacting with and enhancing the function of a global transcriptional repressor, TRIM28. Although TRIM28 has been shown to impact a diverse array of physiologic processes through various molecular mechanisms^52^, our experiments ascribe a novel function to TRIM28, namely its role as an RNA-binding protein. The presence of BORG clearly potentiates the repressive functions of TRIM28 (Fig. 5f,h), implying that the regulatory events downstream of TRIM28 rely in large part upon the lncRNA BORG. It is therefore tempting to speculate that BORG is capable of broadly enhancing the same physiologic and oncogenic processes previously attributed to TRIM28, most notably those essential to driving EMT programs^62^ and expanding cancer stem cell populations in breast cancer^46,47^. Indeed, we recently observed aberrant BORG-TRIM28 complexes to be essential in promoting the self-renewal and expansion of breast cancer stem cells (Gooding & Schiemann, *in preparation*). Along these lines, BORG:TRIM28 complexes are necessary for reprogramming the transcriptional signatures of latent breast cancer cells (Fig. 6d) to align with those associated with a more aggressive and metastatic phenotype (Fig. 6e). Nonetheless, future studies must parse out which loci are specifically regulated by TRIM28 in a BORG-dependent *versus* -independent manner (*i.e*., loci inhibited by TRIM28/KRAB-ZNF co-repressor complexes). Coupled with the genome-wide analyses presented herein, such studies also define the function of TRIM28 as an RNA-binding protein, while simultaneously providing a global picture of candidate loci associated with the suppression or promotion of breast cancer metastasis.

Although our findings have provided novel insights related to the downstream targets and effectors of BORG, we have yet to define the precise mechanisms operant in regulating aberrant BORG expression in developing and progressing mammary tumors. In mice, BORG is expressed in a highly tissue-specific manner, with augmented levels of BORG being detected in the brain, kidneys, and testes, and scarce quantities of this lncRNA being detected elsewhere. BORG expression is dependent upon the presence of multiple paracrine factors, including BMP-2, OP-1^14^, and TGF-β (Fig. 1g), and as such, distinct BORG expression patterns could reflect differences within the stromal composition of each tissue and organ compartment. Similarly, the pro-proliferative functions of BORG are also contingent upon the composition of the cellular microenvironment (Figs. 2d,e), indicating that BORG acts in a highly context-dependent manner. Accordingly, it is tempting to speculate that the frequent augmentation of BORG levels in breast cancer metastases to the brain (Fig. 8e) results from local stromal signals produced by neural tissues that support the upregulation of BORG in CNS metastases to the brain, thereby compelling disseminated breast cancer cells to initiate their metastatic outgrowth. Indeed, owing to the upregulation of BORG observed in response to cellular stresses, these paracrine signals could act in concert with the metabolic and junctional stresses conferred by the metastatic microenvironment to greatly enhance BORG expression at the site of metastasis. However, such a model fails to account for the induction of BORG expression observed in primary lesions of genetically distinct breast cancer subtypes (Fig. 1b,c,d). Nonetheless, the early transforming activities of BORG also display a tissue-specific predisposition (*i.e*.active in breast and inactive in colon; Fig. 1e), suggesting that distinct microenvironments are uniquely permissive to the upregulation of BORG during the malignant transformation of epithelial cells. As such, future studies need to elucidate the precise microenvironmental cues operant in driving aberrant BORG expression in developing human breast cancers.

Advancements in drug development have broadened the scope of targetable effectors in cancer cells to recently include targeting of noncoding RNAs^63^. These advances have led to the development of promising therapies that target oncogenic RNA molecules, such as locked nucleic acid-based antisense oligonucleotides^13,64^ and, more encouragingly, lipid nanoparticle-based RNA-interference approaches that have shown safety and efficacy in preliminary human trials^65^. Despite residing in early phases of development, the potential utility of these methods makes the targeting of oncogenic lncRNAs such as BORG a clinical reality. Accordingly, with inhibition of BORG impeding the development of aggressive tumors, such efforts could prove effective in deterring the metastatic outgrowth of disseminated cells, particularly those persisting in a latent state. Experiments designed to test the therapeutic potential of BORG in preclinical therapy models are currently underway.

## Methods

### Cell culture and constructs

D2.HAN (D2.OR and D2.A1) cells and 4T1 progression series (67NR, 4T07, and 4T1) were obtained from Fred Miller (Wayne State University, Detroit, MI) and grown in DMEM (Sigma-Aldrich) supplemented with 10% FBS and 1% Pen/Strep. MCF-7 and NMuMG cells were acquired from ATCC and grown in DMEM (10% FBS; 1% Pen/Strep) supplemented with 1 μg/ml insulin (Sigma-Aldrich). D2.HAN cells were engineered to stably express firefly luciferase by transfection with pNifty-CMV-luciferase, followed by zeocin selection (500 μg/ml; Invitrogen) as described^33^. The full-length BORG transcript and mutant BORG deletions were created as described^14^ and used to transfect D2.OR cells, followed by selection with G418 (500 μg/ml) to generate stable cell lines. Similarly, doxycycline-controlled expression of BORG was generated *via* VSVG lentiviral co-transduction of D2.OR cells with M2-rTTA vector and vectors expressing BORG under control of the Tet-Response Element (pLVX-tight-puro). Cellular depletion of BORG in D2.A1 cells was achieved by VSVG lentiviral transduction of pGeneClip vectors containing a nonspecific shRNA sequence or those specific for BORG (Promega; Table S2). D2.OR cells were transduced with lentivirus produced from vector containing expression cassettes for both SpCas9 and the chimeric single guide RNA scaffold (pLentiCRISPRv2)^66^, followed by selection with puromycin (5 μg/ml). sgRNA design was carried out using CHOPCHOP design tool^67^ to target various exons of TRIM28 and minimize off target binding of sgRNAs. Genomic DNA was extracted from cells using Quick gDNA Miniprep Kit per manufacturer’s instructions (Zymo Research), and genomic region targeted by specific sgRNAs was PCR amplified and sequenced to assess resulting genomic mutation arising from non-homologous end joining.

### 3D-organotypic culture and outgrowth assays

Longitudinal 3D-outgrowth quantification assays were carried out by plating cells (9000 cells/cm^2^) atop solidified cushions of Cultrex reconstituted basement extract (50 µl/well; 96 well plate; Trevigen), containing 3 mg/ml collagen type I (BD Biosciences) when specified as rigid. Cells were cultured in complete media supplemented with 5% Cultrex, as well as the p38 MAPK inhibitor, SB203580 (10 μM, Calbiochem), or doxycycline (1 μg/ml, Sigma-Aldrich), where indicated. Bioluminescent readings were obtained on designated days by addition of D-luciferin potassium salt (Gold Biotechnology; Cat. No. LUCK), followed by quantification *via* GloMax-Multi detection system (Promega). Initial bioluminescent reading to which subsequent cell growth was normalized was obtained 24 hr after cell plating, and complete media/Cultrex was replaced after every addition of D-luciferin. Longitudinal 3D-outgrowth assays for subsequent immunoblotting were carried out by plating cells (31,500 cells/cm^2^) atop solidified Cultrex cushion (600 µl/well; 6 well plate) and grown in complete media supplemented with 5% Cultrex. For protein and RNA isolation, cells were non-enzymatically isolated using Cultrex 3D-Culture Cell Harvesting Kit (Trevigen) and subsequently prepared for immunoblotting or real-time PCR or microarray analysis, as described below.

### Immunoblotting

Cells isolated from 3D-culture were homogenized on ice in RIPA buffer (50 mM Tris, 150 mM NaCl, 6 mM sodium deoxycholate, 1.0% NP-40, 0.1% SDS, pH 7.4) supplemented with protease inhibitor cocktail (Sigma-Aldrich) and phosphatase inhibitors (10 mM sodium orthovanadate, 40 mM β-glycerophosphate, 20 mM NaF). Lysates were cleared by centrifugation and subjected to immunoblot analysis as described^27^ using antibodies listed in Table S3. For co-immunoprecipitation assays, cells were grown in standard 2D-cultures and solubilized with Buffer H supplemented with 1% Triton X-100^68^, at which point clarified cell extracts (200 µg protein/tube) were immunoprecipitated with anti-TRIM28 antibodies (Thermo Fisher). Immunoprecipitation of sumoylated proteins was carried out on D2.OR cells lysed in 0.15 M Tris-HCl (pH 6.7), 5% SDS, and 30% glycerol supplemented with protease inhibitors as described^69^ and subjected to immunoprecipitation with antibodies for TRIM28, followed by immunoblotting with antibodies against αSUMO2/3 (Cell Signaling Technology).

### Cell cycle analysis

D2.OR derivatives were synchronized by serum starvation prior to plating in standard tissue culture dishes or atop 3D-Cultrex cushions in 12-well plates (400 μl Cultrex/well; 6600 cells/cm^2^). After 5 days, cells were isolated using Cultrex 3D Culture Cell Harvesting Kit and stained using CellSimple Propidium Iodide RNase Staining Solution Kit (Cell Signaling Technology) per the manufacturer’s instructions. Briefly, 2.0 × 10^5^ cells were isolated and stained for 15 min with 0.5 ml PI/RNase solution with 0.05% Triton Cell Lysis Buffer to permeabilize cells. Cells were diluted with PI/RNase Staining Solution to 1.0 × 10^5^ cells/ml prior to quantitative fluorescence analysis with CellSimple Cell Analyzer (Cell Signaling Technology) to assess percentage of cells in each phase of cell cycle. Data was analyzed with FlowJo software to acquire cell cycle distribution curves. PI cell cycle analysis was carried out on cells grown in 2-D culture upon reaching 70% confluency.

### *In vivo* bioluminescence

Firefly-expressing D2.HAN derivatives (1.0 × 10^6^ cells/mouse) were injected into the lateral tail vein of 8-week-old BALB/c mice and pulmonary outgrowth of cells was measured by bioluminescent readings at the stated times post-injection. Briefly, mice were injected with D-luciferin, anesthetized with isofluorane, and imaged 5 min after injection using the IVIS Spectrum (Perkin Elmer) *in vivo* imaging system. Quantification was performed with Living Image software (Xenogen) to determine luminescent flux through the lungs and values were normalized to total luminescence at day 7.

### Anoikis assays

D2.OR derivatives were cultured atop poly-HEMA-coated 96-well plates in reduced serum-media (0.5%) and incubated for 24 h, at which time they were subjected to the Caspase-Glo 3/7 Assay (Promega) per the manufacturer’s protocol to quantify degree of apoptotic cells.

### Tumor dissociation

Mice were sacrificed on day 56 post-injection and lungs were immediately removed, finely minced, and subsequently digested for 2 h at 37 °C under continuous rotation in 6.5 Wünsch units/ml Liberase TM (Roche Life Sciences) dissolved in PBS. After incubation, samples were passed through 70-micron nylon cell strainers and washed twice with PBS prior to plating in complete DMEM medium containing 500 μg/ml zeocin to select for firefly-expressing cells. Colonies of D2.OR cells that arose were collected and subjected to qRT-PCR to quantify BORG expression and 3D-longitudinal outgrowth assays.

### Real-time PCR and gene expression analysis

Cells were isolated from 3D-culture as described above and RNA was extracted using TRIzol reagent (Thermo Fisher) according to the manufacturer’s instructions. For PDX tissue, samples were homogenized in TRIzol reagent (1 ml TRIzol per 100 mg tissue) using a handheld rotor-stator homogenizer (Qiagen) followed by RNA extraction and removal of DNA with DNase I (Invitrogen) treatment. One μg of total RNA was subsequently reverse transcribed with iScript cDNA Synthesis Kit (Bio-Rad) and subjected to semi-quantitative real-time PCR using iQ SYBR Green Supermix (Bio-Rad) as previously described^33^ using primers listed in Table S2. Alternatively, with the aid of the Gene Expression and Genotyping Facility within the Case Comprehensive Cancer Center, isolated RNA was converted to cRNA, labeled, fragmented, and hybridized to GeneChip Mouse Gene 2.0 ST Array (Affymetrix) for comprehensive quantification of ~35,000 coding and non-coding transcripts. Probe intensity normalization and inter-sample quality control was performed using Affymetrix Expression Console software, while gene expression analysis and hierarchical clustering was performed using Affymetrix Transcription Analysis Console and GenePattern software^70^. Similarly, for senescence-specific transcriptional analyses, 500 ng RNA was reverse transcribed using RT^2^ first strand kit (Qiagen), and the resulting cDNA was combined with RT^2^ SYBR Green qPCR Mastermix, and loaded into and quantified by the mouse Cellular Senescence RT^2^ Profiler PCR Array (Qiagen) according to the manufacturer’s protocol (Qiagen).

### Histology and immunohistochemistry

Lungs were excised and fixed in formalin prior to their being embedded in paraffin and cut into 5 μm sections and stained with H&E by the Case Comprehensive Cancer Center’s Tissue Resources Core. Immunohistochemical analysis was performed as previously described^68^ using antibodies for Ki-67 or IgG (Table S3).

### Chromatin Immunoprecipitation

ChIP assays were performed according to Abcam X-ChIP protocol with minor modifications. Briefly, 3D-ChIP experiments were conducted on D2.OR cells pooled from 4 wells of a 6-well plate containing 3D-Cultrex cushions (~5 million cells). Cells were immediately fixed in 0.75% paraformaldehyde upon extraction by Cultrex 3D Culture Cell Harvesting Kit. Chromatin shearing was performed by sonicating the samples at 5 watts for 30 sec, followed by 1 min intervals with samples on ice. This procedure was repeated for 28 cycles to yield optimized DNA fragments ranging from 200 bp-1000 bp in length. Twenty-five μg of DNA was used in each IP, which also contained antibodies for TRIM28 (5 μg/IP) or Pol II IP (5 μg/IP) (Table S3). Elution was performed at 65 °C with occasional vortexing for 1 hr. Crosslinks were reversed *via* overnight incubation at 65 °C after RNase A and Proteinase K treatment, and DNA was isolated with Qiagen PCR purification kit. qPCR of ChIP-enriched DNA was performed as described above and both percent genomic input and fold enrichment compared to beads only control were calculated for each sample. Pausing index was calculated by dividing the fold enrichment of Pol II at the gene body versus its fold enrichment at the transcriptional start site.

### RNA immunoprecipitation

RIP assays were performed as described previously^71^ with slight modifications. Briefly, ~1.5 × 10^7^ D2.OR cells were fixed in 1% paraformaldehyde for 10 min followed by quenching with 200 mM glycine for 5 min. Cells were collected, washed twice with ice-cold PBS, and resuspended in 0.2X PBS/20% nuclear isolation buffer (1.28 M sucrose; 40 mM Tris-HCl, pH 7.5; 20 mM MgCl_2_; 4% Triton X-100), followed by frequent mixing on ice for 20 min. Nuclei were pelleted by centrifugation at 2,500 g for 15 min, resuspended in 1 ml RIP buffer (150 mM KCl; 25 mM Tris, pH 7.4; 5 mM EDTA; 0.5 mM DTT; 0.5% NP40; protease inhibitor cocktail [Sigma]; and 100 U/ml RNaseOUT Ribonuclease Inhibitor [Invitrogen]), then sonicated on ice for two 15 sec cycles at 5 watts. Cellular debris was pelleted at 13,000 rpm for 10 min, and supernatants were collected and pre-cleared with protein A beads (pre-washed with RIP buffer) with rotation for 1 hr at 4 °C. Lysates were collected and incubated at 4 °C overnight with antibodies specific to TRIM28 or RelA/p65 (Table S3; 5 μg each), followed by incubation with protein A beads at 4 °C for 1 hr. Beads were subsequently pelleted and washed twice with RIP buffer, once with FA500 buffer (1 mM EDTA pH 8.0, 50 mM HEPES-KOH, pH 7.5; 500 mM NaCl; 0.1% sodium deoxycholate; 1% Triton X-100; 100 U/ml RNaseOUT Ribonuclease Inhibitor), once in LiCl wash buffer (1 mM EDTA, pH 8.0; 250 mM LiCl; 0.5% NP-40; 0.1% sodium deoxycholate; 10 mM Tris, pH 8.0; 100 U/ml RNaseOUT Ribonuclease Inhibitor), and once in TE buffer. Beads were collected, and captured protein:RNA complexes were eluted in RIP elution buffer (10 mM EDTA; 1% SDS; 100 mM Tris, pH 8.0, 100 U/ml RNaseOUT Ribonuclease Inhibitor). Crosslink reversal was carried out *via* Proteinase K treatment for 1 hr at 42 °C, followed by 1 hr incubation at 65 °C prior to extracting RNA using TRIzol reagent, as above. DNA was removed with DNase I treatment (Invitrogen) and RNA was subjected to semi-quantitative real-time PCR as described above.

### Statistics

Unless otherwise stated, two-tailed Student’s *T-*tests were performed on *n* ≥ 3 biological replicates, where *P* < 0.05 was considered significant.

### Study approval

All animal experimental protocols were approved by the Institutional Animal Care and Use Committees for Case Western Reserve University, and all animal studies herein were performed in accordance with these approved protocols. All studies involving human samples were performed in accordance with the Case Western Reserve University Institutional Review Board (IRB), specifically pertaining to UHCMC IRB Number: 01-13-43 C. Informed consent was received from all patients in these studies and all identifying information was redacted.

### Data Availability

Microarray datasets generated in this study have been deposited in GEO under accession number GSE99234. Further datasets generated during and/or analyzed during the current study are available from the corresponding author on reasonable request.

## Electronic supplementary material

Supplementary Information
