# Supplementary material for: The lncRNA BORG Drives Breast Cancer Metastasis and Disease Recurrence
Source: Sci Rep. 2017 Oct 5;7:12698. doi: 10.1038/s41598-017-12716-6 (PMC5629202; doi:10.1038/s41598-017-12716-6)
Supplement: Supplementary file 1 — Supplementary Information [file 41598_2017_12716_MOESM1_ESM.pdf]

SUPPLEMENTAL MATERIALS

The lncRNA BORG Drives Breast Cancer Metastasis and Disease Recurrence

Alex J. Gooding[1], Bing Zhang[2], Fereshteh Kenari Jahanbani[2], Hannah L. Gilmore[3], Jenny C. Chang[4], Saba Valadkhan[2,#] and William P. Schiemann[1,#]

Author Affiliations: [1]Case Comprehensive Cancer Center, Case Western Reserve University, Cleveland, OH 44106. [2]Department of Molecular Biology and Microbiology, Case Western Reserve University, Cleveland, OH 44106. [3]Department of Pathology, University Hospitals, Case Medical Center and Case Western Reserve University, Cleveland, OH 44106. [4]Houston Methodist Research Center, Houston, TX 77030.

[#]Corresponding Authors:
William P. Schiemann, Case Comprehensive Cancer Center, Case Western Reserve University, Wolstein Research Building, 2103 Cornell Road, Cleveland, OH 44106 Phone: 216-368-5763. Fax: 216-368-1166. E-mail: william.schiemann@case.edu

Saba Valadkhan, Department of Molecular Biology and Microbiology, Case Western Reserve University, Wood Building, 10900 Euclid Avenue, Cleveland, OH 44106 Phone: 216-368-1068. Fax: 216-368-3914. E-mail: saba.valadkhan@case.edu

Running Title: BORG Drives Breast Cancer Metastasis and Recurrence

Conflict of Interest: The authors have declared that no conflict of interest exists.

Table S1. Pathoclinical Features of Patients From Which PDX Tissue was Derived[58].

Xenograft Line	Intrinsic Subtype	Tumor Source	Xenograft Metastasis Rate	Patient Metastatic Site	Patient Nodal Status	Patient Clinical Response
BCM-3613	HER2+/ER-/PR-	Plural Fluid; Post**	23.8%	Brain	nr	<30%
BCM-4664	TNBC	Primary; Pre*	0%	None	+	<30%
BCM-3963	HER2+/ER-/PR-	Primary; Pre*	13.6%	Brain	+	≥30%
BCM-4013	TNBC	Primary; Pre*	21.4%	None	+	<30%
BCM-2665	TNBC	Primary; Post**	7.1%	None	-	<30%
BCM-4913	TNBC	Primary; Post**	0%	None	+	<30%
BCM-3887	TNBC	Recurrence; Pre*	14.3%	Brain	-	<30%
BCM-3204	TNBC	Primary; Pre*	28.6%	None	-	<30%

*Pre, pre-treatment; **Post, post-treatment; nr, not reported

Table S2. Oligonucleotides

Target	Application	Sequence (5' to 3')
GAPDH (human)	PCR-Sense	5'- TGCACCACCAACTGCTTAGC
GAPDH (human)	PCR-Antisense	5'- GGCATGGACTGTGGTCATGAG
GAPDH (mouse)	PCR-Sense	5'- CAACTTTGGCATTGTGGAAGG GCTC
GAPDH (mouse)	PCR-Antisense	5'- AGTAGAGGCAGGGATGATGTT
BORG (mouse)	PCR-Sense	5'- AAATCCCAGCAGCCGTGTAA
BORG (mouse)	PCR-Antisense	5'- GTCTTTGCTCACTTCCCCCA
BORG Del1	PCR-Sense	5'- GGCTGTCAACGCTGTAGTCT
BORG Del1	PCR-Antisense	5'- CCAGCTCTTCTGCCAGTCAA
TRIM28 sgRNA 1	CRISPR-sense	5'- GCTCTCACAGAACAGCACGA
TRIM28 sgRNA 2	CRISPR-sense	5'- CCAGCGGGTGAAATACACCA
TRIM28 sgRNA 3	CRISPR-sense	5'- CGCCGCAGCGAATAATTCGG
TRIM28 sgRNA 4	CRISPR-sense	5'- GTGCTACTCCAAAGACATCG
TRIM28 sgRNA 5	CRISPR-sense	5'- CGTGTGAATGGCGGCCTCGG
TRIM28	PCR-Sense (genomic sequencing)	5'- GTCCGGCTGCTTCCTCAG
TRIM28	PCR-Antisense (genomic sequencing)	5'- CCAGACACTGAGACCGCATA
p21	PCR-sense	5'- CCGTGGACAGTGAGCAGTTG
p21	PCR-antisense	5'- TGGGCACTTCAGGGTTTTCT
gadd45a	PCR-sense	5'- GAGCAGAAGACCGAAAGGATG
gadd45a	PCR-antisense	5'- GTGAAATGGATCTGCAGAGCC
p21	ChIP qPCR-sense (TSS)	5'- CGCTGCGTGACAAGAGAATA
p21	ChIP qPCR-antisense (TSS)	5'- CCTCCCCTCTGGGAATCTAA
p21	ChIP qPCR-sense (Gene Body)	5'- GTCTGAACTCCAGGACCACG
p21	ChIP qPCR-antisense (Gene Body)	5'- CATTGGCAACCAGAAGCGAG
gadd45a	ChIP qPCR-sense (TSS)	5'- CTTTCCGCTCAACTCTGCCT
gadd45a	ChIP qPCR-antisense (TSS)	5'- GCAGTCAGGCCATTGGGTTA
gadd45a	ChIP qPCR-sense (Gene Body)	5'- GTGTGCTGGTGACGGTAAGA
gadd45a	ChIP qPCR-antisense (Gene Body)	5'- CACGCTCCGTACCCTATCAC
Chmp2a	PCR-sense	5'- TCGCTTAATCCGGAAACGGC
Chmp2a	PCR-antisense	5'- ACAACCGTCAGAACTAGCGGA
Zbtb45	PCR-sense	5'- GCGTGAGGATCGATGGCAG
Zbtb45	PCR-antisense	5'- CATCACAGAAGTGACCGCCT
BORG (human)	PCR-sense (PDX)	5'- CGATGGGTCACATGACAAAG
BORG (human)	PCR-antisense (PDX)	5'- TTCTCCTGGGGGAAAAATAGA
BORG (human)	PCR-sense (Brain Metastasis)	5'- GCCATATGCCTTGTGACTTGC
BORG (human)	PCR-antisense (Brain	5'- CCTCCTAACCCTGCAATTTAACC

	Metastasis)	
BORG (mouse)	shBORG (#1)	5'- GATCTCGCGTTGACAGTGAGCGA TAGGCCATGCTCCAGATATTATAGTGA AGCCACAGATGTATAATATCTGGAGC ATGGCCTAGTGCCTACTGCCTCGA
BORG (mouse)	shBORG (#2)	5'- GATCTCGCGTTGACAGTGAGCGC GCCTCTCTCCTCGATAAAGAGTAGTG AAGCCACAGATGTACTCTTTATCGAG GAGAGAGGCATGCCTACTGCCTCGA

Table S3. Antibodies

Antibody	Dilution	Supplier (catalog #)
Ki-67	1:100	BD Biosciences (#550609)
IgG (Rabbit)	5 μg (IP)	Cell Signaling Technology (#2729)
IgG (Mouse)	1:100 (IHC)	Santa Cruz Biotechnology (sc-2025)
SUMO-2/3	1:500	Cell Signaling Technology (#4971)
TRIM28	5 μg (ChIP & IP)	ThermoFisher (#PA5-27648)
TRIM28	1:1000 (Western Blot)	Abcam (#ab2253)
RelA	5 μg (IP)	Cell Signaling Technology (#8242)
Actin	1:10000	Sigma-Aldrich (#A5441)
Kid-1/Pim-3	1:500	Cell Signaling Technology (#4165)
Cleaved Caspase 8	1:500	Cell Signaling Technology (#9429)
Phospho-p38 MAPK	1:500	Cell Signaling Technology (#9211)
Phospho-ERK1/2	1:1000	Cell Signaling Technology (#9101)
Total p38α MAPK	1:1000	Santa Cruz Biotechnology (sc-728)
Total ERK1/2	1:1000	Cell Signaling Technology (#4695)
Pol II	5 μg (IP)	Santa Cruz Biotechnology (sc-899)

Supplementary Fig. 1: Gooding et al.

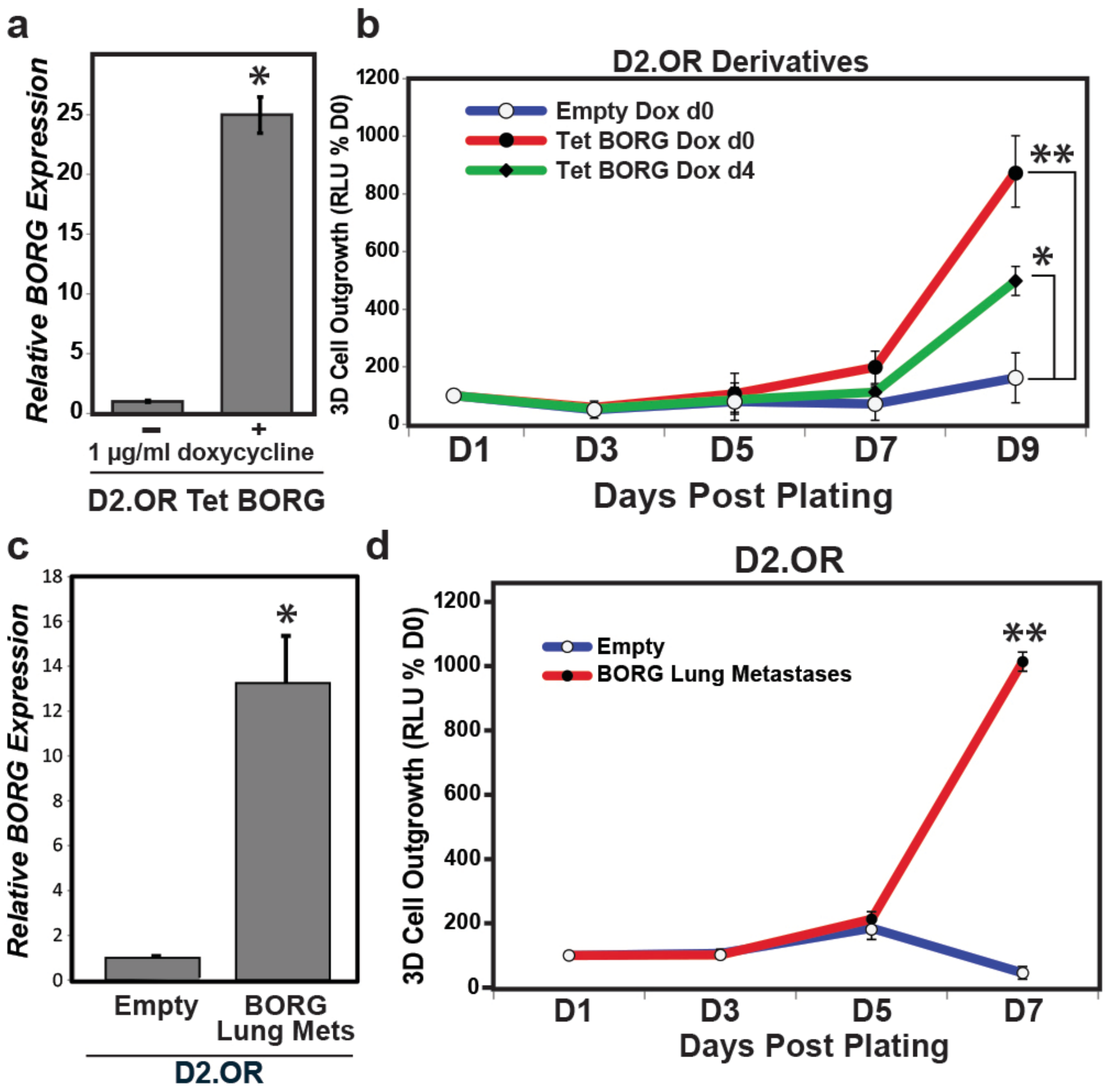

Supplementary Fig. 1. Inducible BORG expression promotes 3D outgrowth; lung metastases maintain BORG-induced phenotypes *ex vivo*. (**a** and **b**) Doxycycline-inducible (1 µg/ml; (**a**)) expression of BORG at either day 0 (Dox d0) or day 4 (Dox d4) significantly stimulated D2.OR organoid growth in 3D-cultures as determined by longitudinal bioluminescent assays ((**b**); mean ± SEM; **P*<0.05; ***P*<0.01). (**c**) Lungs harboring metastases were dissociated and propagated on tissue-culture plates to isolate and expand metastatic cells, which expressed significantly increased levels of BORG (**P* < 0.05). (**d**) BORG-expressing D2.OR cells isolated from the lungs of mice and cultured *ex vivo* retain proliferative phenotypes as compared to their parental counterparts maintained in standard *in vitro* culture (***P* < 0.01).

Supplementary Fig. 2: Gooding et al.

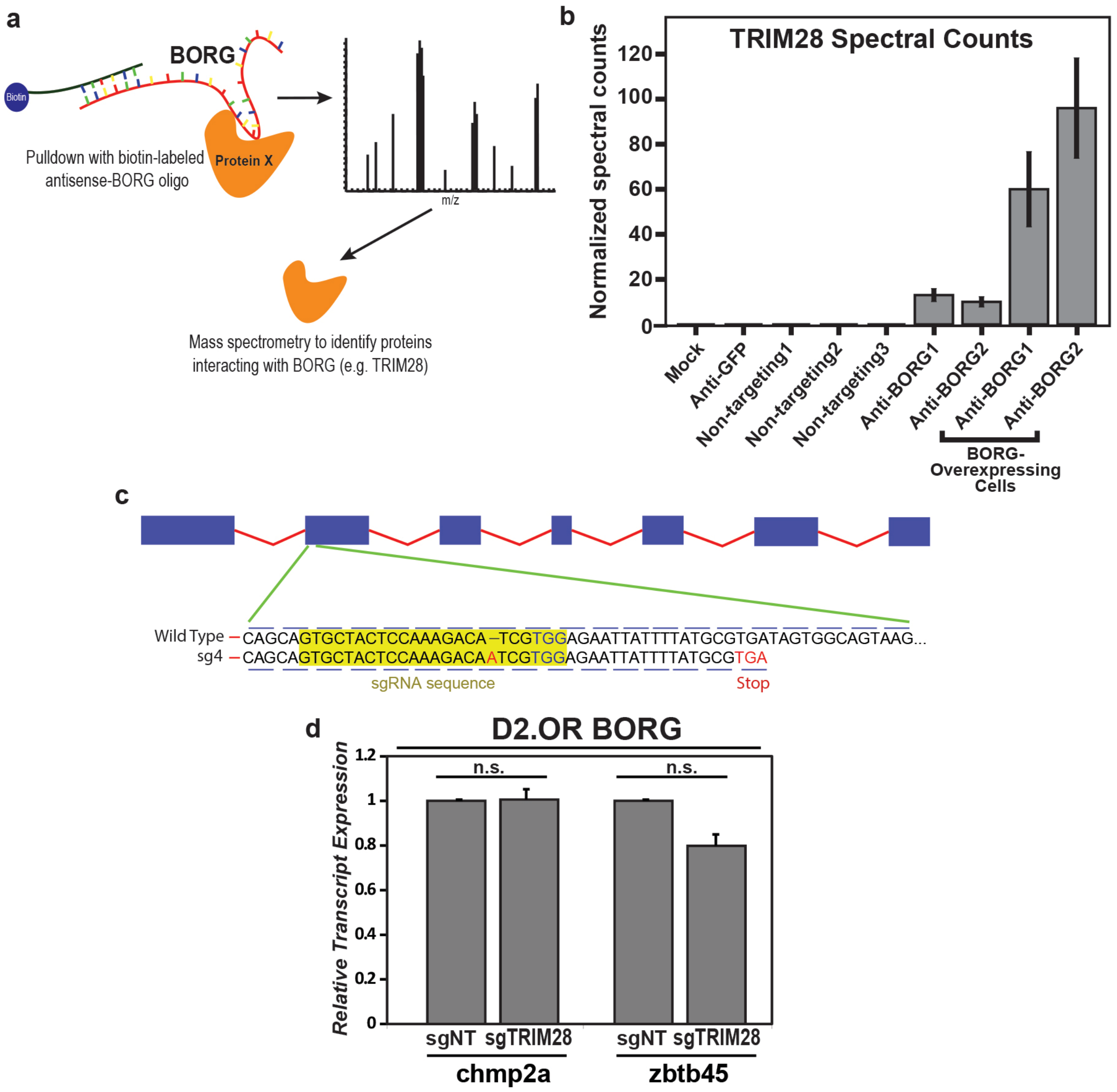

Supplementary Fig. 2. CRISPR/Cas9-mediated knockout of BORG-binding partner identified *via* mass spectrometric analysis. (**a**) Schematic representation of the method used to capture and identify BORG-interacting proteins. (**b**) Whole cell extracts prepared from parental and BORG-expressing C2C12 myoblasts were incubated with biotin-labeled anti-BORG probes, which were captured by addition of streptavidin-agarose beads. Proteins bound to BORG were identified by mass-spectrometry. Shown are the mean (± SD; n=2) TRIM28 spectral counts normalized to input material. Mock sample lacked anti-BORG oligonucleotides. (**c**) Single guide RNA 4 (sg4) targeted the proximal segment of the second exon of TRIM28 and produced a single nucleotide insertion, thereby producing a frameshift and premature stop codon that generated a truncated TRIM28 variant. (**d**) qRT-PCR analysis of genes bordering the TRIM28 locus (*chmp2a* and *zbtb45*) revealed no significant alteration in transcript abundance, demonstrating that CRISPR/Cas9 targeting did not result in substantial genomic deletions or disruptions at the targeted locus.

Supplementary Fig. 3: Gooding et al.

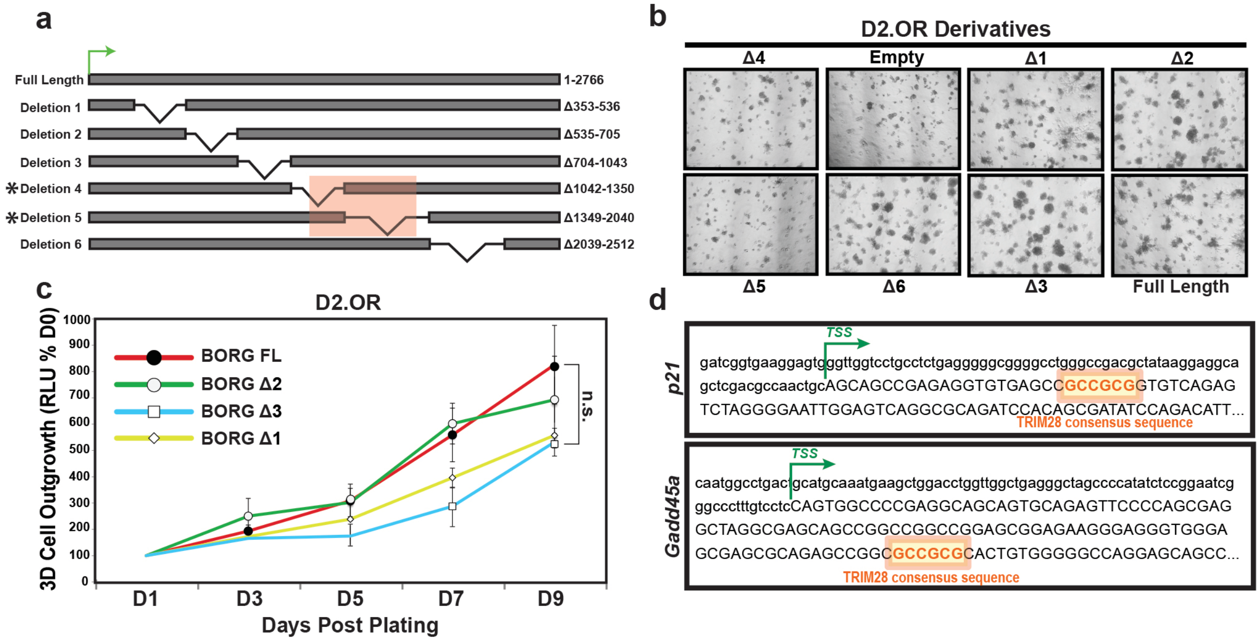

Supplementary Fig. 3. Identification of BORG deletion mutants that mediate its coupling to TRIM28 and the emergence from metastatic latency. (**a**) Schematic depicting the location and size of internal deletions in full-length BORG. Shaded box represents approximate segment of BORG necessary for TRIM28 interaction; asterisks represent deletions that partially ablated TRIM28-binding of BORG. (**b** and **c**) D2.OR derivatives expressing either full-length or mutant BORG transcripts were grown longitudinally in 3D-culture. Shown are representative photomicrographs of organoids formed by these D2.OR derivatives after 7 days (100X; **b**). Longitudinal outgrowth was quantified *via* bioluminescence. Data are the mean (±SEM; **c**). (**d**) Schematic depicting TRIM28 consensus binding sequence (GCCGCG) just distal to transcription start sites (TSS) of both *p21* and *gadd45a* loci (25 and 103 bases downstream of *p21* and *gadd45a* TSS, respectively).

Supplementary Fig. 4: Gooding et al

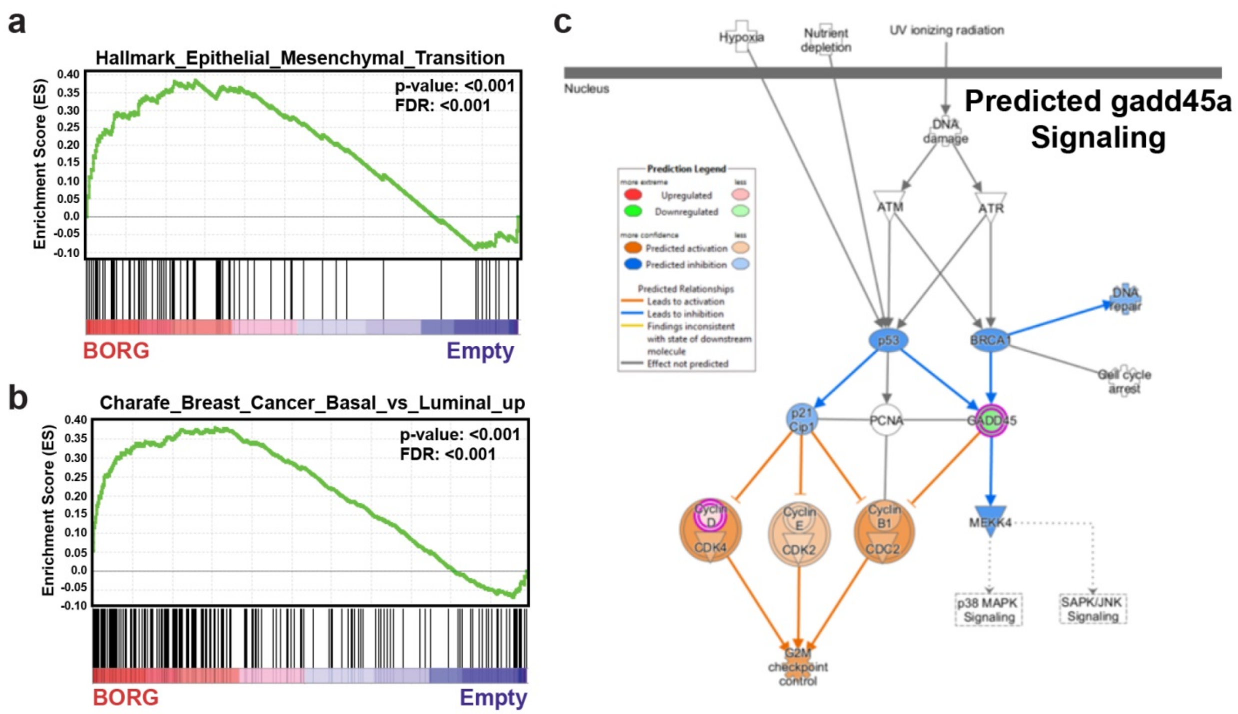

Supplementary Fig. 4. Transcriptional signatures regulated by BORG in latent D2.OR cells. (**a**) GSEA demonstrated enrichment of the hallmark EMT gene signature in D2.OR-BORG cells *versus* their parental counterparts. (**b**) D2.OR-BORG cells were enriched in gene signatures associated with aggressive basal breast cancer lesions *versus* indolent luminal lesions. (**c**) IPA identified a *gadd45a*- and *p21*-dependent increase in G2/M checkpoint progression in D2.OR-BORG cells *versus* parental counterparts.

Supplementary Fig. 5: Gooding et al

```
human           AGCAGGAGCGATGGGTCACATGACAAAGAGAGAAGCAGGTTTTCTCTCCACATCTGATAA
chimp           AGCAGGAGCGATGGGTCACATGACAAAGAGAGAAGCAGGTTTTCTCTCCACATCTGATAA
macaca          AGCAGGATCGATAGGTCACATGACAAAGAGAGAAGCAGGTATTCTCTCCACATCTGATAA
cow             -------------------------------------GGTTTTCTCTCTACATTAAATAA
pig             ---------GATGGCTCAGGTGACCTAGAGAGAAGCGGCTTTTCTCTCTACCTGAAGTCA
cat             ------------------------------------------------------------
dog             ------------------------------------------------------------
mouse           AGCGGGAACCAGGGCTCCTATGACAGAAA-AGAAGCAGGC-TTCTCTCTGCAATTCATAA
rat             AGCAGGAACCAGAGCTCTTCTGACAGAAA-AGAAACAGGC-TTCTCTCTGCGTTTCATAG

human           ACACTGACAAGAGTTAGGTGATTTTCCTACACACAGCACTATCTTGTTCTGAGAAAGGAT
chimp           ACACTGACAAGAGTTAGGTGATTTTCCTACACACAGCACTATCTTGTTCTGAGAAAGGAT
macaca          ACACCGACAAGAGTTAGGTGATTCTCCTACACATGGCACTATCTTGTTCTGAGAAAGGAT
cow             ACACTAACAAGAGTTAGGTGATCCCCCT-CACCCTACACTTCCTTGTTCAGAGAAAGGAT
pig             ATGCTGACAAGAGTTGGGTGATCTTCCTACACCCACCGCTGTCTGGTTCAGAGGAAGGGT
cat             ------------------------------------------------------------
dog             -------------------------------------------TTGTTCTGAGGAAGGAT
mouse           ACACCGACAGGAATATGTTGCTTTTCCTAAGCCCTTCGCTGTCTTTCCAAGAAGAATTTT
rat             ACACTGACAAGAGTCGGATGCTTTTCCTAGGCCCTGCGCTGCCTTTCCAAGAAGAATCTT

human           TTG--CCTTAACCAAATACGGGGCCTGACAGCTTTTTCTCTCAAATGAATCAGTGGGGAG
chimp           TTG--CCTTAACCAAATACGGGGCCTGACAGCTTTTTCTCTCAAATGAATCAGTGGGGAG
macaca          TTG--CCTCAACCAAATACAGGGCCTGACAGCTTTT-CTCTCAAATGAATCAGTGGGGAG
 cow             CTG--ACTTCACCAGACATAAAACCTGACAGGTATTTCTCGCGAATGAATCAGTGGGGAG
pig             TTG--ACTTTACCAAATAGAAAACCTGACAGGTAGGCCTCCCCAGTGAATCAATGGGGTG
cat             -----------------------------------TTCTCCCAAATGAGTCAGTGGGGAG
dog             CTG--ACTCAACCA-ATACAGAACCTGACA--TATCTCTCTCAAATGAATCAGTGGGGAG
mouse           TTTGTCCTTAACCAAATGTGAAAT---GCACATTTTGAGCACAAA-GAATCAAAGCTCCA
rat             TTCATCTTTAACCAAATAGGAA------CTGCTGGTGCGCATAAATGAATGAATGCTTCA
                                                           *

human           GAGCTGAAGTGACTTTTTGTTTTG---AAAGAAATGCAATATTTAAAGAGAGA------G
chimp           GAGCTGAAGTGACTTTTTGTTTTG---AAAGAAATGCAATATTTAAAGAGAGA------G
macaca          GAGCTGAAGTGACTTTTTGTTTTG---AAAGACATGAAATATTTAAAGAGAGA------G
cow             GACCTGAAGCGACTTAGTTTTTTATCTAAAGAAATGTAATATTTAAAGAAAGA------G
pig             GAGCTGAAGTGACTTTTTTTTAAATTTAAAGAAATGTAATATTTAAAGA----------G
cat             GAGCTGAAATGGCAGTTGTTTAAGA--AAAGGAGTGTAATATTTAAAGAGAGT------T
dog             GAGCTCAAATGACATTTGGGGGGGG--AAAGAAATGTAATATTTAAAGAGA--------G
mouse           ACAGCAAACTGACATGACATCCTTTA-CAGGAAGTAAAATATTTAAAGACAGAGTCAGAG
rat             ACAGCAGGCTGATGCGACATCCTTTA-AAGGAAGAGAAATATTTAAAGACAGA------G
                          *                 * *      ************

human           GTTTCTATTTTTCCCC-CA-GGAGAAGGGAGCCCTTAATATTCCCATCAACGATGACACA
chimp           GTTTCTATTTTTCCCC-CA-GGAGAAGGGAGCCCTTAATATTCCCATCAACGATGACACA
macaca          TTTTCTATTTTTCCCC-TA-GGAGAAGGGAGCCCTTAATATTCCCATCAACGATGACACG
cow             TTCGCTATCCCCCCTC-TTTGGGGAAGGGAGCCCTTAATTTTCCCATCAACTCTGACACA
pig             TTTTCTATTCCCCCTC-CTTGGGGAAGAGAGCCCTTAATTTTCCCATCAACTCTGACATG
cat             TTCTGCTTCCCCCTCG-CTTGGGGAAAGGAGCCCTTAATATTCCCATCAACCGTGACACG
dog             TTCTCAGTCCCCCCTCGCTTGGGGAAGGGAGCCCTTAATCTTCCCATCAAC---------
mouse           TCTTCTATTCCCCTCC-CCCCAGGAAGAAACCCTTTAATATTCCCATTAACCTTGA----
rat             TCTTCTATTTTCCCCC---TGAGGAAGAAACCCTGTAATATTCCTATTAACCTTGACATG
                                                   **** **** ** ***

human           TGTGAATCACAGCCCTTTCTT-TCCTATAAAAGCA-----TCATGATTCAACACAAAGCC
chimp           TGTGAATCACAGCCCTTTCTT-TCCTATAAAAGCA-----TCATGATTCAACACAAAGCC
macaca          TATGAATCACAGCCCTTTCTT-TCCTATAAAAGCA-----TCATGATTCATTACAAAGCC
cow             TGTGAATCACAGCACTTTCAG-TCCTATAAAAGCC-----TCATGATTCATCAACAACCC
pig             TGTGAATCACAGCCCTTCTGT-TCCTATAAAAGCA-----TCATGATTCATCAGCAGCCT
cat             TGTGAATCACACCCCTTTCGC-TCTTACAGAAGCG-----GCATGATTCATCGCGAACCC
dog             ------------------------------------------------------------
mouse           TGTGAATCACAGCGCTCCTTC-TTCCCTGTAGGCAGATAATCATGACTCACCACAGACCC
rat             TGTGAATCACTGAGCTCTTTCATCCCCTGTAGGTAGATTATCATGACTCACCACAGACCC

human           CGCC----AGCTGTGGGCTGCATGTGATTCT----
chimp           CGCC----AGCTGTGGGCTGCATGTGATTCT----
macaca          CGCC----AGCTGTGGGCTGCATGTGATTCT----
cow             TTCT----AGCTGTGGGCTGC--------------
pig             TGCC----AGCTGTGGGCTGCA-------------
cat             TGCCCGCCAGCTGTGGGTTGCGTGTTATTCT----
dog             -----------------------------------
mouse           CT------GACTGTGGGCTTCATGTAATTCTGGCA
rat             AC------GACTGTGGGCTTCATGTAACCCT----
```

Supplementary Fig. 5. Phylogenetic alignment of the conserved domain of BORG. Consistent with the majority of lncRNAs, BORG contains domains harboring significant primary

sequence conservation. Depicted is the alignment of one such domain between multiple mammalian species. Domain consists of a conserved region in the third exon of BORG, which encompasses nucleotides 2140 to 2515 of mouse BORG. Sequence changes compared to human BORG are highlighted in color. Asterisks mark two clusters of invariant nucleotides within this region.

Supplementary Fig. 6: Gooding et al

Full unedited gel for Figure 3D

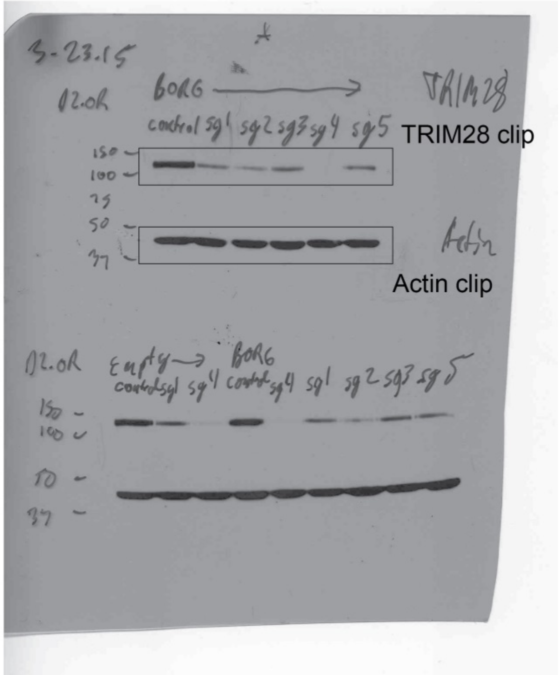

Full unedited gel for Figure 4G

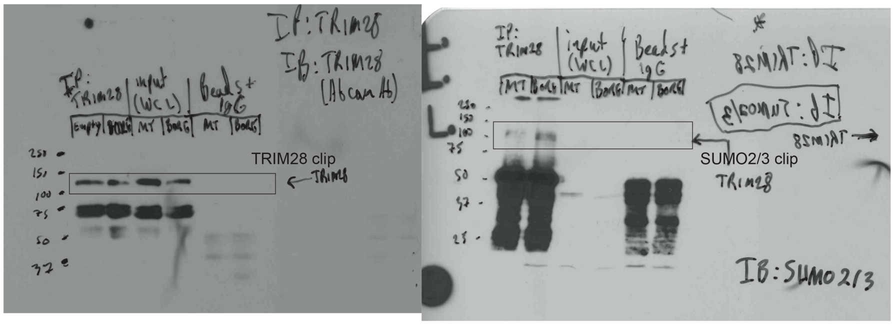

Full unedited gel for Figure 4H

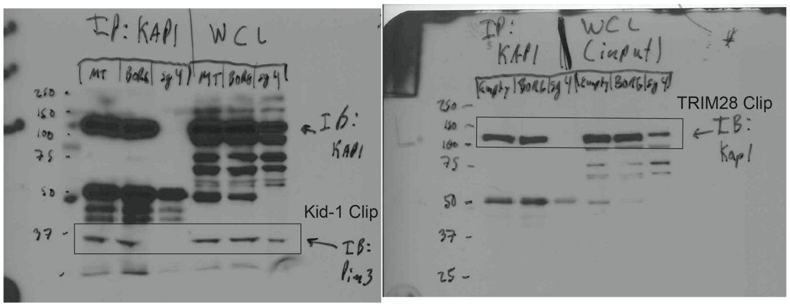

Full unedited gel for Figure 6B

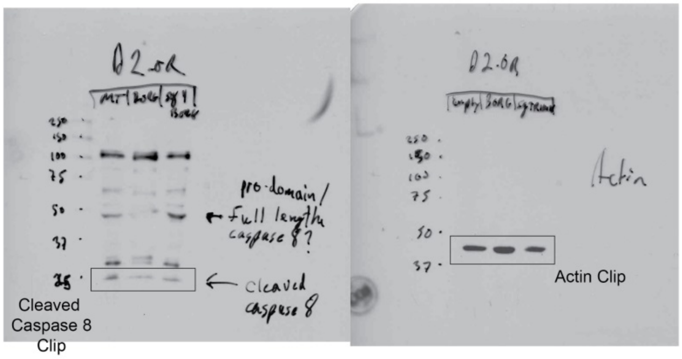

Full unedited gel for Figure 6C

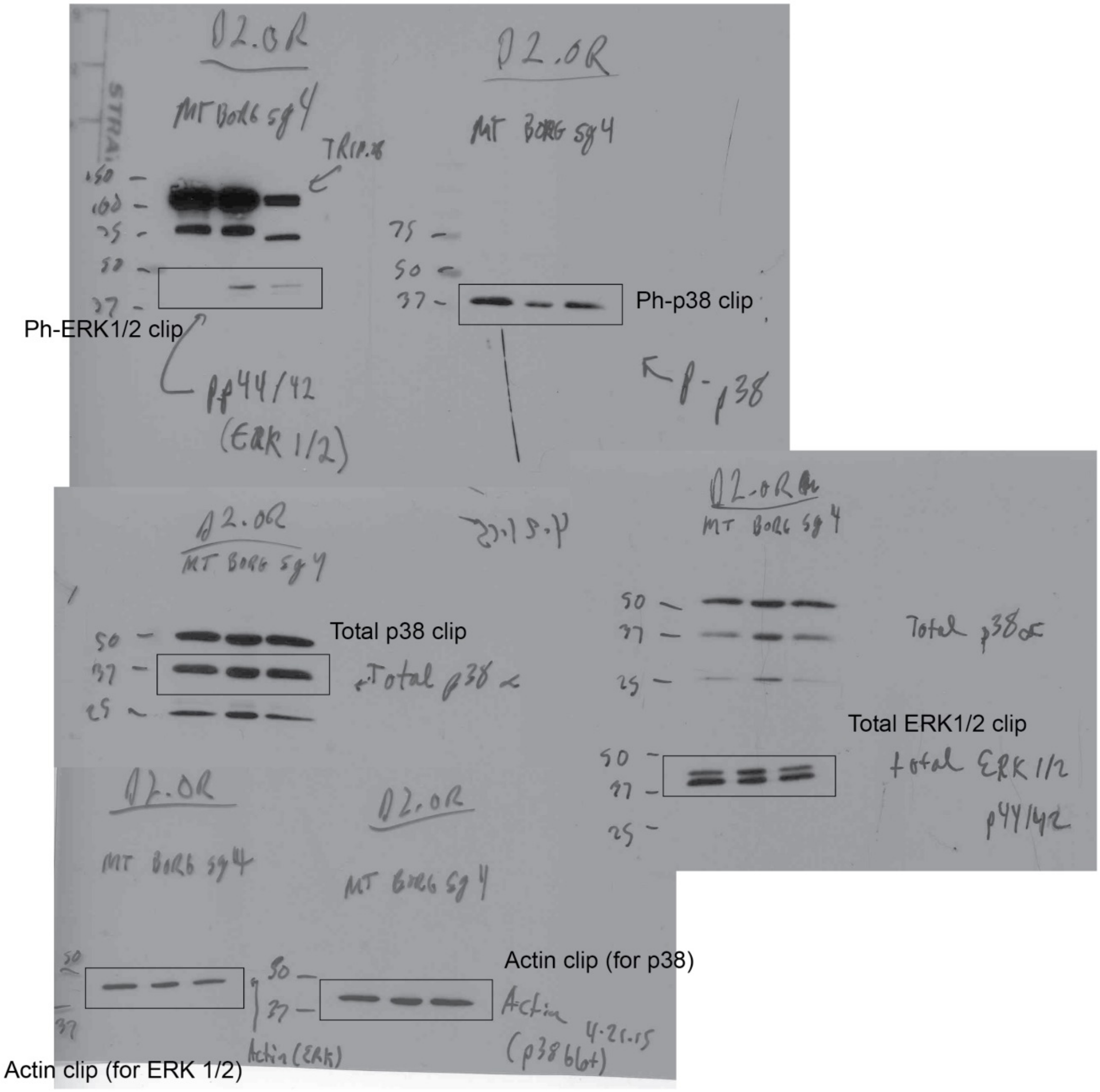

Full unedited gel for Figure 6D

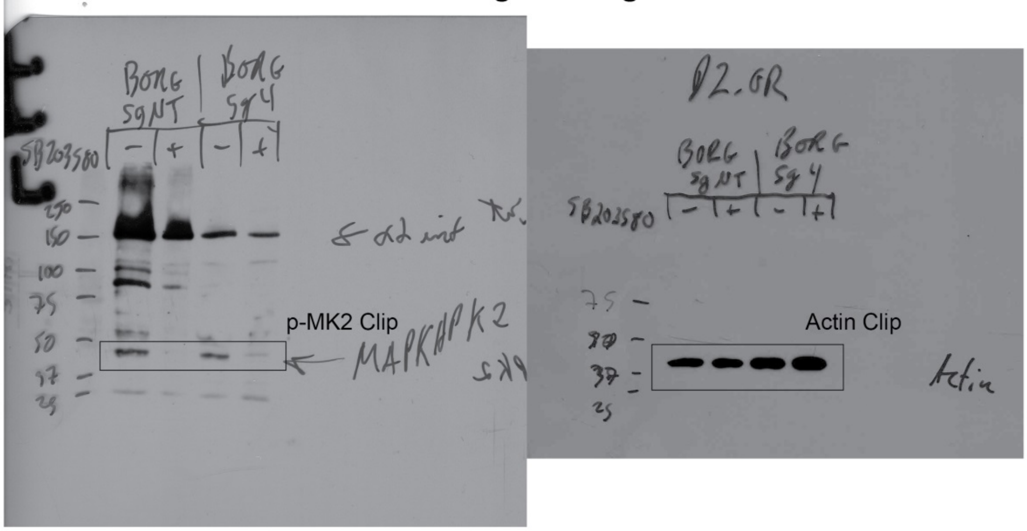

Supplementary Fig. 6. Full, unedited blots depicting cropped blots throughout text.
